# A Strategy for the Acquisition and Analysis of Image-Based Phenome in Rice during the Whole Growth Period

**DOI:** 10.34133/plantphenomics.0058

**Published:** 2023-06-08

**Authors:** Zhixin Tang, Zhuo Chen, Yuan Gao, Ruxian Xue, Zedong Geng, Qingyun Bu, Yanyan Wang, Xiaoqian Chen, Yuqiang Jiang, Fan Chen, Wanneng Yang, Weijuan Hu

**Affiliations:** ^1^ Institute of Genetics and Developmental Biology, Chinese Academy of Sciences, Beijing 100101, China.; ^2^ National Key Laboratory of Crop Genetic Improvement, National Center of Plant Gene Research (Wuhan), Hubei Hongshan Laboratory, Huazhong Agricultural University, Wuhan 430070, China.; ^3^ Northeast Institute of Geography and Agroecology, Key Laboratory of Soybean Molecular Design Breeding, Chinese Academy of Sciences, Harbin 150081, China.

## Abstract

As one of the most widely grown crops in the world, rice is not only a staple food but also a source of calorie intake for more than half of the world’s population, occupying an important position in China’s agricultural production. Thus, determining the inner potential connections between the genetic mechanisms and phenotypes of rice using dynamic analyses with high-throughput, nondestructive, and accurate methods based on high-throughput crop phenotyping facilities associated with rice genetics and breeding research is of vital importance. In this work, we developed a strategy for acquiring and analyzing 58 image-based traits (i-traits) during the whole growth period of rice. Up to 84.8% of the phenotypic variance of the rice yield could be explained by these i-traits. A total of 285 putative quantitative trait loci (QTLs) were detected for the i-traits, and principal components analysis was applied on the basis of the i-traits in the temporal and organ dimensions, in combination with a genome-wide association study that also isolated QTLs. Moreover, the differences among the different population structures and breeding regions of rice with regard to its phenotypic traits demonstrated good environmental adaptability, and the crop growth and development model also showed high inosculation in terms of the breeding-region latitude. In summary, the strategy developed here for the acquisition and analysis of image-based rice phenomes can provide a new approach and a different thinking direction for the extraction and analysis of crop phenotypes across the whole growth period and can thus be useful for future genetic improvements in rice.

## Introduction

Rice (*Oryza sativa* L.) is one of the most widely grown crops and is a staple food for over half the population throughout the world, with consumption increasing dramatically in recent years; rice crops have contributed importantly to food security [1–3]. Enhancing the process of breeding elite varieties with increased yields, high qualities, and strong disease resistance is critical to ensure the safety of rice production and to satisfy the global food supply–demand balance while facing the challenge of feeding a rapidly growing population under the background of dramatic climate change [4,5].

With the development of molecular biology, the advent of next-generation technology and high-density single-nucleotide polymorphism (SNP) genotyping technologies have greatly propelled rice function genomics studies [6–8]. With the rapid accumulation of a large amount of rice genome resequencing data, exploring how to link genotypes with phenotypes is a prerequisite for precise rice breeding design [6,9]. However, traditional phenotyping methods that rely on manual methods are time-consuming, costly, and have poor repeatability in plants and are thus far behind the development of genotyping; in addition, destructive methods in general cannot obtain the continuous measurements or observations of the dynamic development process and are unable to meet the actual needs of breeding [9]. The lag of the innovative technological development of plant phenotyping has indeed become a bottleneck in crop breeding research and development.

Recently, high-throughput plant phenotyping platforms [10–12] that are highly efficient, nondestructive, and practical have appeared as a solution in crop breeding research to bridge the developmental gap between genotype and phenotype analyses of crops [13,14]. In previous studies, some high-throughput plant phenotyping platforms, including the Scanalyzer series, rice automatic phenotyping platform (RAP), or other platforms, have been applied in crop plants, such as maize, rice, wheat, and sorghum [15–19]. Using commercial software or open-source image analysis pipelines, many image-based traits (i-traits) and dynamic shoot growth characteristics were nondestructively quantified, thus displaying the unique advantages of this method and the potential for further developments in crop genetics and breeding studies [18,20].

It has been widely recognized that the dynamic plant growth and development processes can be evaluated on the basis of high-throughput phenotyping facilities. The approaches and standards for quantifying plant phenotypes have gradually attracted much attention from breeding researchers. In recent years, with the emergence of innovative artificial intelligence methods, such as machine learning, which has been widely applied in the image process of phenotyping analysis, remarkable achievements have been made because of the powerful adaptability and image processing capabilities of such methods [21–23]. At present, customizing and designing phenotype analysis pipelines with suitable image-processing algorithms for different morphological structures of plants is the basis for exploring observable, quantifiable phenotypes and for improving the accuracy and efficiency of phenotype analyses.

In this study, we developed a strategy for the acquisition and analysis of i-traits in rice based on a high-throughput visible light imaging platform during the whole growth period. We extracted the rice phenotypic traits at the individual plant, panicle, culm, and grain levels of crops and further analyzed these traits at the temporal scale to obtain organ–time i-traits in rice during the whole growth period to explore the potential association between phenotype and genotype. We found that these i-traits could be used as good predictors of the final yield, and multiple differences among rice genotypes of different population structures and breeding regions of i-traits were also illustrated. Combined with genome-wide association studies (GWAS), we also demonstrate that further analyses performed in organ–time dimensions based on traits obtained by high-throughput phenotyping have potential as a feasible research direction in the fields of genomics and rice breeding.

## Material and Methods

### Plant materials and experimental design

In our study, 93 rice accessions, including 32 indica and 61 japonica rice accessions with 4 replications, were planted in a greenhouse located at the Institute of Genetics and Developmental Biology, Chinese Academy of Sciences in Beijing. Specific information on the rice accessions is shown in Table S1. All rice accessions (4 replications per accession) were screened across all growth stages (every ~7 d, from 42 to 182 d after sowing). The growth conditions were recorded as follows: seed germination lasting approximately 24 h (at 25 to 32 °C), airing buds above 20 h (at 20 °C), and then transplantation into a greenhouse in Changping. Fertilizer was applied at sowing with 2 g of compound fertilizer and 3 kg of soil per pot. Eight grain-related traits were determined by a yield traits scorer (YTS-RICE-4D, Wuhan GreenPheno Co. Ltd., China). In addition, to evaluate the measurement accuracy, the culm dry weight, panicle dry weight, and individual plant dry weight were also measured destructively after harvest.

### Image analysis and trait extraction

**Fig. 1.**
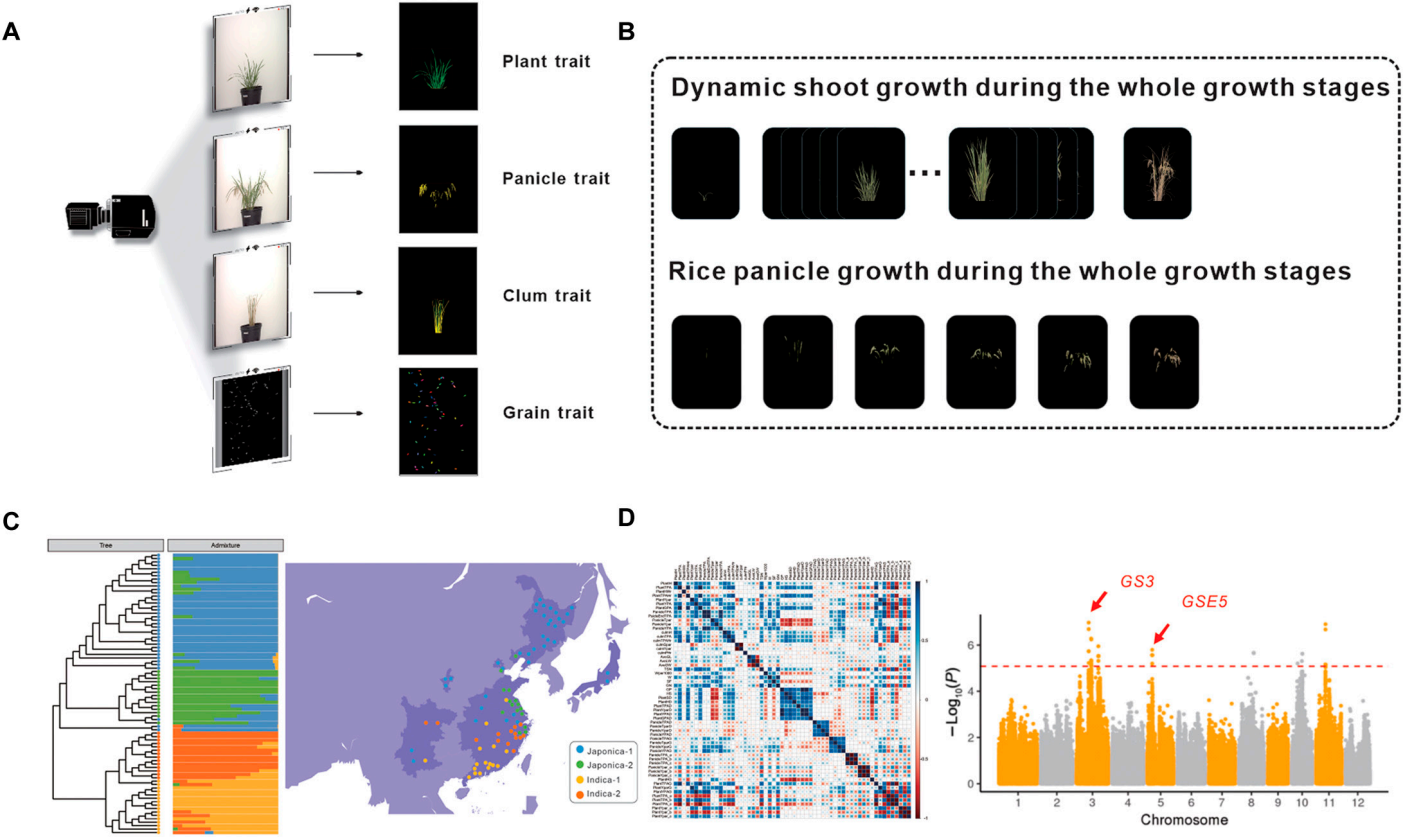
High-throughput rice plant phenotyping results. (A) Images of rice acquisitions. (B) Dynamic shoot growth during all growth stages. (C) Group clustering and regional division of different rice genotypes. (D) Data analysis of i-traits.

The phenotyping facility used in this study (ScanLyzer, LemnaTec GmbH, Germany) captured side-view RGB images of growing plants and culm images after the panicles were harvested (Fig. 1A and B). An image analysis pipeline of i-traits in rice [18] during the whole growth period was used to obtain 7 plant-related traits, 10 plant growth-related traits, and 6 culm-related traits. Using the deep learning network of SegNet [24] to segment the rice panicles, 5 panicle-related traits and 10 panicle growth-related traits were also obtained. With these plant and panicle traits observed during all growth stages (Fig. 1B), 12 phenological traits were also analyzed (Table). The experimental arrangement of the rice material is shown in Fig. S1, the variation in phenological traits is exemplified in Fig. S2, and the dynamic processing method is shown in Movie S1.

**Table.** Definitions of extracted image-based traits. **a**, **b**, and **c** represented coefficients of quadratic polynomials.

Organ–temporal dimensions	Character classification	Abbreviation	Trait definition
Organ dimension	Plant-related traits	PlantH	Height of the whole plant
		PlantTPA	Total projected area of the whole plant
		PlantHWr	Ratio of the height and width of the whole plant
		PlantTPAHr	Ratio of the total projected area and height of whole plant
		PlantYpar	Ratio of the yellow projected area and total projected area of the whole plant
		PlantYPA	Yellow projected area of the whole plant
		PlantGPA	Green projected area of the whole plant
	Panicle-related traits	PanicleTPA	Total projected area of panicles
		PaicleEndTPA	Value of the final total projected area of panicles
		PanicleTpar	Ratio of the projected panicle area and whole plant area
		PanicleYpar	Ratio of the yellow projected area and total projected area of panicles
		PanicleYPA	Yellow projected area of panicles
	Culm-related traits	culmH	Height of the plant culm
		culmTPA	Total projected area of the plant culm
		culmTPAHr	Ratio of the projected area and height of the plant culm
		culmGpar	Ratio of the green projected area and total projected area of the plant culm
		culmYpar	Ratio of the yellow projected area and total projected area of the plant culm
		culmPHr	Ratio of heights of the culm and whole plant
	Grain-related traits	AveGL	Mean value of the grain length
		AveLW	Mean value of the grain length/width ratio
		AveGW	Mean value of the grain width
		TFN	Total spikelet number
		Wper1000	1,000-grain weight
		W	Yield per plant (filled grain weight)
		SF	Spikelet fertility
		GN	Filled grain number
Temporal dimension	Phenological traits	GP	Days to harvest from sowing
		HS	Days to the beginning of heading from sowing
		PlantSD	Days to the onset of plant senescence from sowing
		PlantHD	Days to the maximum whole-plant height from sowing
		PlantTPAD	Days to the maximum total projected whole-plant area from sowing
		PlantYparD	Days to the maximum ratio of the yellow projected area to the total projected area of the whole plant from sowing
		PlantYPAD	Days to the maximum whole-plant yellow projected area from sowing
		PlantGPAD	Days to the maximum green projected area of the whole plant from sowing
		PanicleTPAD	Days to the maximum total projected area of the whole plant from heading
		PanicleTparD	Days to the maximum ratio of the projected panicle and whole-plant areas from heading
		PanicleYparD	Days to the maximum ratio of the yellow projected area to total projected area of panicles from heading
		PanicleYPAD	Days to the maximum yellow projected area of panicles from heading
	Panicle growth-related traits	PanicleTPAG	Value of daily growth of the total projected panicle area
		PanicleTparG	Value of daily growth of the ratio of the projected areas of panicles and the whole plant
		PanicleYparG	Value of daily growth of the ratio of the yellow projected area and total projected area of panicles
		PanicleYPAG	Value of daily growth of the yellow projected area of panicles
		PanicleTPA_a	TPA_panicle = **a**t^2^ + bt + c
		PanicleTPA_b	TPA_panicle = at^2^ + **b**t + c
		PanicleTPA_c	TPA_panicle = at^2^ + bt + **c**
		PanicleYpar_a	ypar_panicle = **a**t^2^ + bt + c
		PanicleYpar_b	ypar_panicle = at^2^ + **b**t + c
		PanicleYpar_c	ypar_panicle = at^2^ + bt + **c**
	Plant growth-related traits	PlantHG	Daily growth value of the height of the whole plant
		PlantTPAG	Daily growth value of the total projected area of the whole plant
		PlantYparG	Daily growth value of the ratio of the yellow projected area to the total projected area of the whole plant
		PlantYPAG	Daily growth value of the yellow projected area of the plant
		PlantTPA_a	TPA_plant = **a**t^2^ + bt + c
		PlantTPA_b	TPA_plant = at^2^ + **b**t + c
		PlantTPA_c	TPA_plant = at^2^ + bt + **c**
		PlantYpar_a	ypar_plant = **a**t^2^+bt+c
		PlantYpar_b	ypar_plant = at^2^ + **b**t + c
		PlantYpar_c	ypar_plant = at^2^ + bt + **c**

### Biomass modeling and performance evaluation of the phenotyping platform

The dry weights of the culm, panicle, and aboveground shoot parts were measured destructively after harvest. With the total projected areas of 3 different organs—culmTPA (total projected area of the plant culm), PanicleTPA (Total projected area of the panicles), and PlantTPA (total projected area of the whole plant)—in the last shoots before harvest, 6 models (including linear, quadratic, exponential, power, Gaussian, and triangular sine models) were applied to obtain the predicted dry weight of the culm, panicle, and aboveground shoot parts. The model accuracy was evaluated by comparing the coefficient of determination (*R*^2^), mean absolute percentage error (MAPE), root mean square error (RMSE), and mean absolute error (MAE) between the predicted and actual dry weights. A 5-fold cross-validation approach [25] was also used to test the prediction accuracy by assessing the *R*^2^, MAPE, RMSE, and MAE values. Statistical analyses of the 6 models were performed with LabVIEW 2015 (National Instruments, USA) and Python 3.8. The detailed statistical analysis results are shown in Tables S2 to S7, and related technical documentation is also shown in Note S1.

### Growth-related trait extraction

To determine the best plant growth model based on 2 plant-related traits, the PlantTPA and the ratio of the yellow projected area to the PlantTPA (PlantYpar), 6 models (including linear, quadratic, exponential, power, Gaussian, and sine triangular models) were evaluated. Similarly, 2 panicle-related traits, including the PanicleTPA and the ratio of the yellow projected area to the PanicleTPA (PanicleYpar), were used to determine the best panicle growth model. The 6 models were implemented using LabVIEW 2015 (National Instruments, USA) and MATLAB 2018b (MathWorks, USA), and related technical documentation is also shown in Note S1. On the basis of the best growth model, 6 plant growth-related traits and 6 panicle growth-related traits could be derived (Table 1).

### Grain yield prediction using phenotypic traits

With all these i-traits considered in 6 different trait groups (plant-related trait group, panicle-related trait group, culm-related trait group, phenological trait group, panicle growth-related trait group, and plant growth-related trait group; Table 1), linear stepwise regression was used to evaluate the variance explained in terms of the yield. The linear stepwise regression analysis was implemented with IBM SPSS statistic software (IBM Corp., USA) with the stepping method criterion. Variables were added into the model or removed compared to the preset criterion of setting 0.05 and 0.1 as the entry and removal values of the use probability of *F*, respectively, and related technical documentation is also shown in Note S1.

### Correlation analysis between grain-related and other phenotypic traits

To assess the correlation relationship between grain-related traits and other phenotypic traits during all growth stages, 7 trait matrices were prepared and examined using the Mantel test [26]. We computed the pairwise distances between samples on the basis of the grain-related trait group and 6 other trait groups (the plant-related trait group, panicle-related trait group, culm-related trait group, phenological trait group, panicle growth-related trait group, and plant growth-related trait group). We used Euclidean distances for the data to obtain distance matrices and computed partial Mantel correlations between the grain-related traits and 6 trait groups (9,999 permutations) based on the vegan R software package (R Foundation for Statistical Computing, Austria).

### Kruskal–Wallis test and pairwise multiple comparisons

To compare all the i-traits among the different rice population structures and breeding regions, the Kruskal–Wallis test was used to determine whether the independent groups based on different classification criteria were the same or different. When the value of the Kruskal–Wallis test was calculated as statistically significant (*P* < 0.05), pairwise multiple comparisons among the groups were further performed to locate the source of significance with the Wilcoxon test for at least one of the compared groups that differed from the others. The Kruskal–Wallis test and Wilcoxon test were implemented on the ggsignif R software package (R Foundation for Statistical Computing, Austria), and related technical documentation is also shown in Note S1.

### Principal components analysis

Principal components analysis (PCA) was performed using the correlation matrix of trait values in the pca R software packages (R Foundation for Statistical Computing, Austria) by setting “scale = TRUE.” Twenty-six traits, including 7 plant-related traits, 5 panicle-related traits, 6 culm-related traits, and 8 grain-related traits, were used to compute the PCs (Organ_PC). Furthermore, 32 traits, including 10 plant growth-related traits, 10 panicle growth-related traits, and 12 phenological traits, were used to compute the PCs (Time_PC). The first 2 Organ_PCs and the first 2 Time_PCs explained 63.5% and 58.9% of the total variance in the organ and temporal dimensions, respectively, and were retained for GWAS. GWAS was performed as described subsequently. The detailed statistical analysis results of PCA are shown in Tables S8 and S9, and related technical documentation is also shown in Note S1.

### Genome-wide association study

Sequencing data of rice accessions were mapped to the rice reference genome Nipponbare (IRGSP 1.0) [27] with BWA [28]. Reads with mapping qualities lower than 30 and potential polymerase chain reaction duplicates were removed with SAMtools [29]. Potential misaligned reads caused by insertions/deletions were realigned with GATK [30]. Then, nucleotide variants were called and filtered using the UnifiedGenotyper and VariantFiltration tools in GATK. Biallelic SNPs with missing rates less than 0.1 and minor allele frequencies over 0.05 were used for the GWAS. GWAS was performed using EMMAX [31] with a mixed linear model, and the results were visualized with the R package ggplot2. A neighbor-joining tree was constructed with the same SNPs as GWAS using the R package ‘ape’ [32] and visualized with the R package ‘ggtree’ [33].

## Results

### Panicle segmentation and biomass modeling

After the side-view RGB image was acquired, the rice panicle was segmented with SegNet. A total of 154 panicle images were manually labeled and used as the training set, 40 images were used as the validation set, and 10 images were selected randomly as the testing set to evaluate the segmentation performance with 4 indicators, including the intersection over union (IoU), precision, recall, and F-measure. The detailed segmentation evaluation results of the 10 randomly selected images are listed in Table S10, showing that the mean IoU, precision, recall, and F-measure values were 0.83, 0.89, 0.92, and 0.91, respectively. The manual segmentation results extracted using Adobe Photoshop 2021 (Adobe, USA) and the automatic segmentation results of the 3 rice varieties are shown in Fig. S3.

**Fig. 2.**
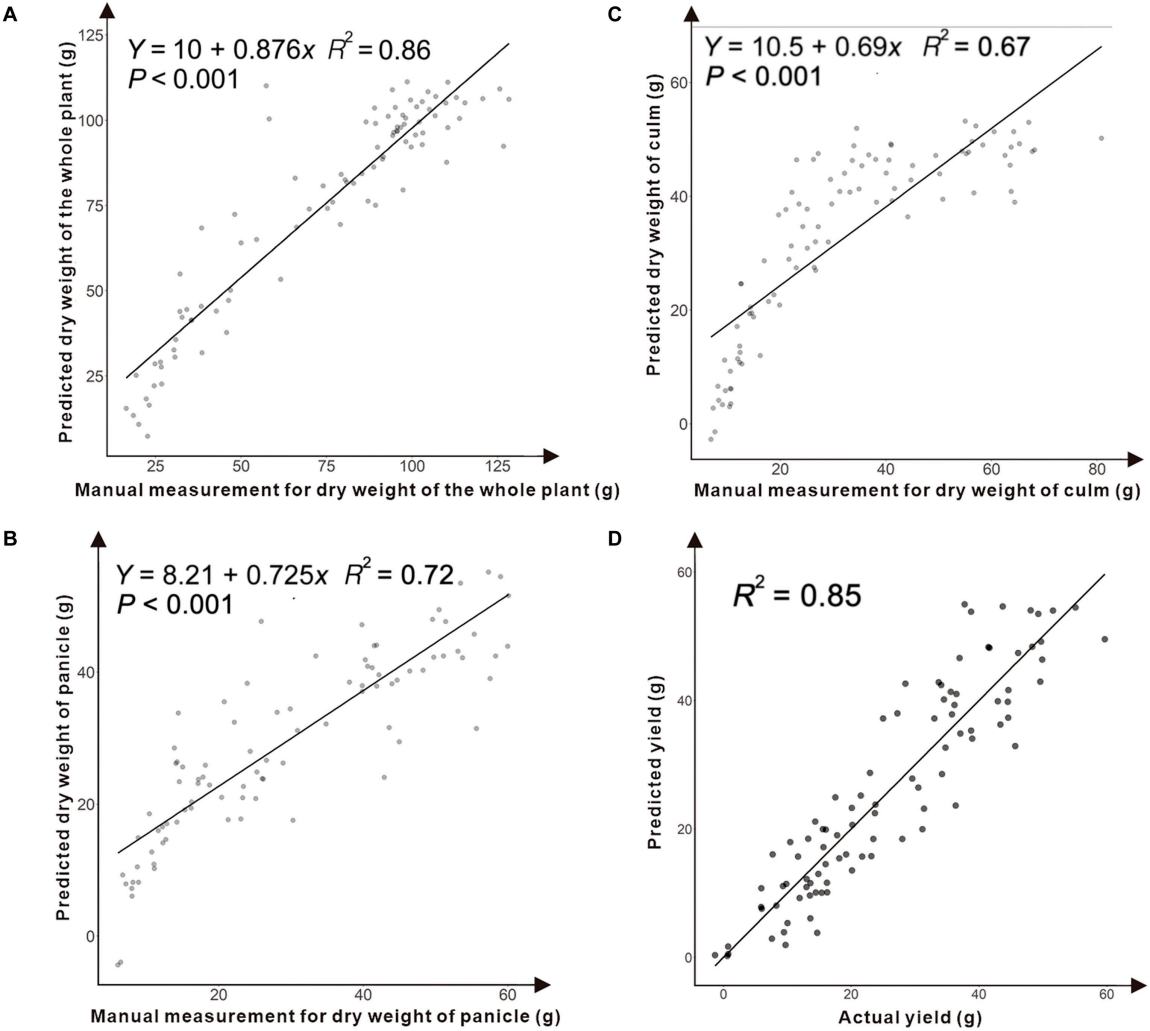
Correlations between manual measurements and automatic measurements. (A to C) Scatterplot of automatic measurements versus manual measurements for whole plant, panicle, and culm separately. (D) Scatterplot of yield predictions with all i-trait groups.

To model the final panicle biomass, we further manually measured the dry weight of the panicle after harvest, and 6 models (including linear, quadratic, exponential, power, Gaussian, and sine triangular models) were compared using the PanicleTPA in the last shoot before harvest. Compared to other models, the quadratic model had a higher *R*^2^ value and lower values of other indices in the panicle dry weight modeling results, and the average *R*^2^ value between the predicted and manual dry weights was 0.72 (Fig. 2B). A detailed statistical summary of the 6 models is shown in Table S2, and the model accuracy was also tested by a 5-fold cross-validation approach (Table S3).

After the dry weight of the culm and the dry weight of the whole plant were obtained after harvest, the 6 models were compared using the culmTPA and PlantTPA, and similar results were found: The quadratic model had a higher *R*^2^ value. The average *R*^2^ values between the predicted dry weight and manually measured dry weight of the whole plant and culm were 0.86 (Fig. 2A) and 0.67 (Fig. 2C), respectively. Detailed statistical summaries of the 6 models for culm biomass and plant biomass are shown in Tables S4 and S6. The model accuracy in terms of the culm biomass and plant biomass was also tested by a 5-fold cross-validation approach, as shown in Tables S5 and S7, respectively.

### Temporal–organ developmental trait extraction

Exploring the growth and senescence trends of plants and panicles would be meaningful and interesting for rice breeding. On the basis of PlantTPA, PlantYpar, PanicleTPA, and PanicleYpar during all growth stages, 6 growth models representing plant growth, plant senescence, panicle growth, and panicle maturation were applied and compared (Tables S11 to S14). It was proven that the quadratic model fitted the trend curve best, with a relatively high *R*^2^ value and low MAPE and SD_APE_ values. The *R*^2^ values of the plant growth, plant senescence, panicle growth, and panicle maturation models were 0.944, 0.916, 0.846, and 0.95, respectively. On the basis of the quadratic model, 6 panicle growth-related traits and 6 plant growth-related traits were derived (Table 1) and can reflect the dynamic changes in plant growth or panicle maturation. For example, PlantTPA_a reflected the growth speed and biomass (Fig. S4A) and PanicleYpar_a reflected the maturation speed (Fig. S4B).

### Yield prediction using i-traits

**Fig. 3.**
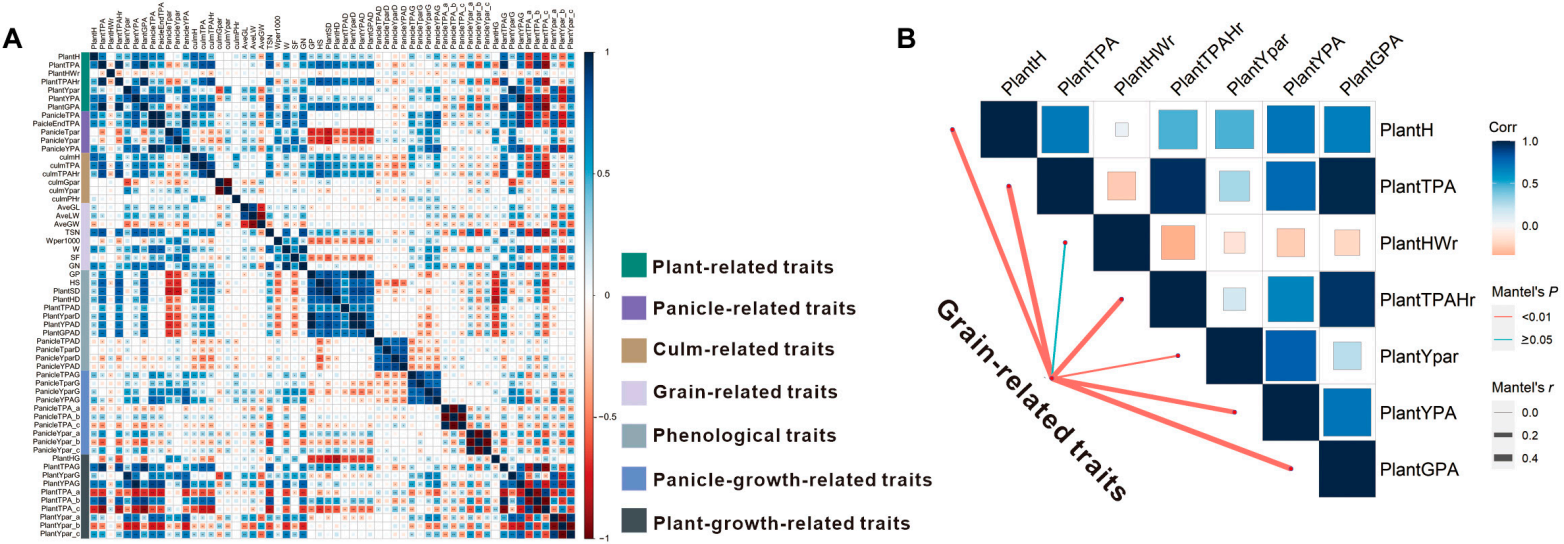
Correlation analysis results based on i-traits. (A) Correlation analysis results of all i-traits. (B) Results of the Mantel tests between the grain-related traits and plant-related traits.

It would be worthwhile for plant breeders to explore whether the existing traits obtained in i-traits can be used to predict the final rice yield. In this study, the rice yield was modeled using 6 groups of i-traits (the plant-related trait group, panicle-related trait group, culm-related trait group, phenological trait group, panicle growth-related trait group, and plant growth-related trait group; Table 1) with linear stepwise regression. The results showed that the explanatory ability of the yield variance reached 84.8% for the 8 traits (Fig. 2D). Compared to the other 5 groups, up to 65% of the phenotypic variance in yield could be explained by the panicle growth-related trait group, which performed better than the other groups. The specific details of these results are shown in Tables S15 to S20. The selected traits based on the i-trait group displayed some interesting features about the rice yield. For example, the maximum value of the total projected panicle area showed a strong positive correlation (PanicleTPA; *r* = 0.804), indicating that the final rice yield would be influenced by the total projected area. Positive correlations were also found between the yield and some growth-related traits, such as PanicleYpar_a (*r* = 0.522), which would reflect a changing panicle maturity pattern and, thus, influence the final yield. The correlations between all the i-traits are shown in Fig. 3, and the results of the Mantel test are shown in Table S21.

### Variations in phenotypic traits with different latitudes

**Fig. 4.**
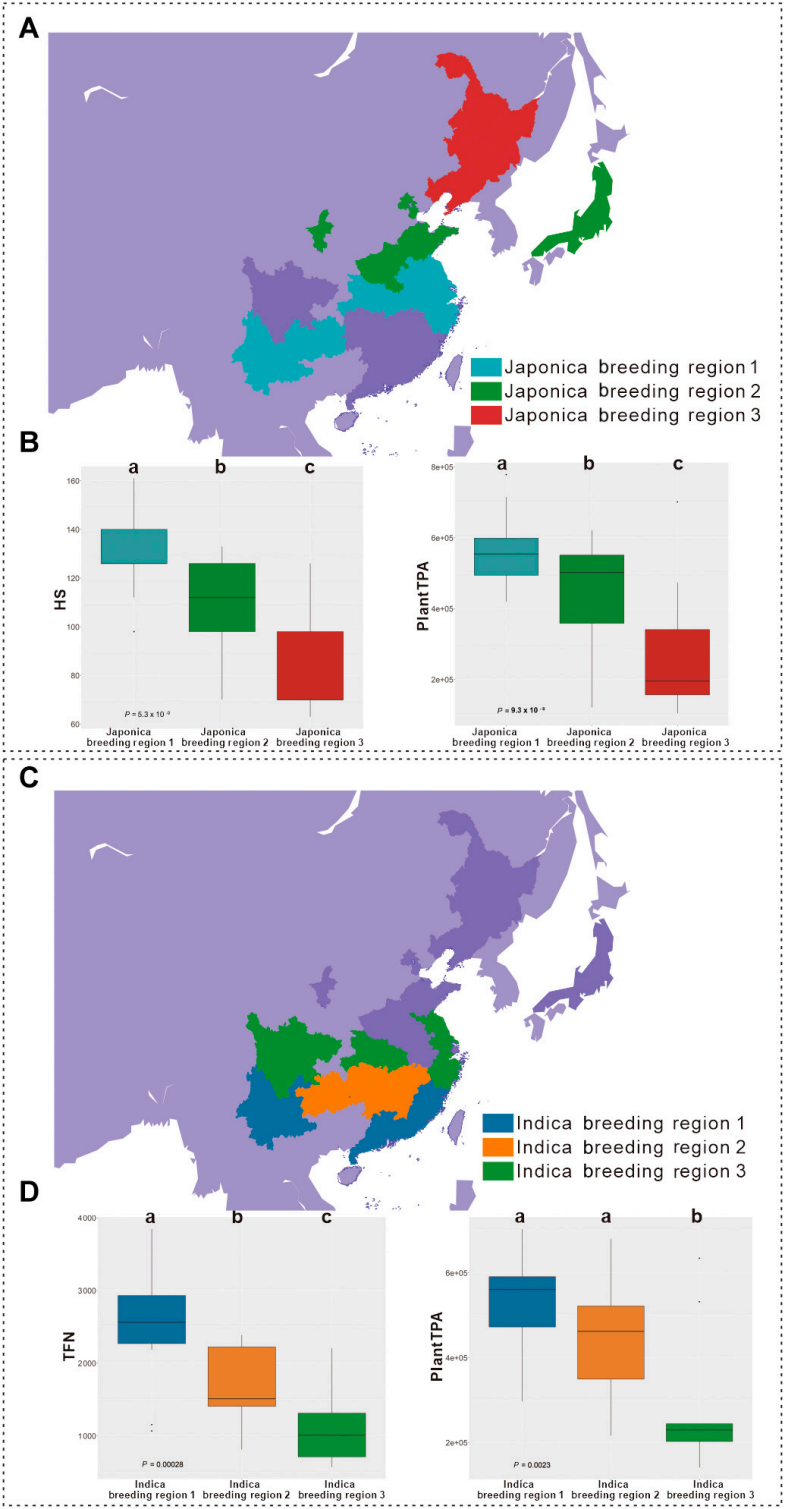
Division of breeding regions and distribution of phenotypes in indica rice and japonica rice. (A) Division of the breeding regions of japonica rice varieties at different latitudes. (B) Distribution of the HS and PlantTPA values of japonica rice in breeding regions at different latitudes. (C) Division of the breeding regions of indica rice varieties at different latitudes. (D) Distribution of the TFN and PlantTPA values of indica rice in breeding regions at different latitudes.

The specific climate conditions of breeding regions necessitate crop varieties with good environmental adaptability, and crop growth and development models also show high inosculation with the latitude of the breeding region [34]. Here, the breeding region was divided into different latitude levels (Fig. 4A and C) to analyze whether an adaptation between the phenotypic traits of the elite rice cultivars and regional latitude was present. Overall, among japonica rice varieties, the heading stage (HS), which greatly contributes to the climatic and regional adaptability of japonica rice, showed a significant negative correlation with the regional latitude (*P* < 0.001) [35], as did the plant biomass (PlantTPA; *P* < 0.001) (Fig. 4B). These results indicated that, among the 61 analyzed japonica accessions, the rice accessions with higher latitudes had earlier HSs and larger plant biomasses. Among the indica rice cultivars, significant differences were also observed between the latitude and total spikelet number (TFN; *P* < 0.001) and between latitude and the plant total projected area (PlantTPA; *P* < 0.001). These results indicated that, among these 32 indica accessions, the higher-latitude rice accessions had smaller spikelet numbers and larger plant biomasses (Fig. 4D).

### Variation in phenotypic traits with different rice genotypes

**Fig. 5.**
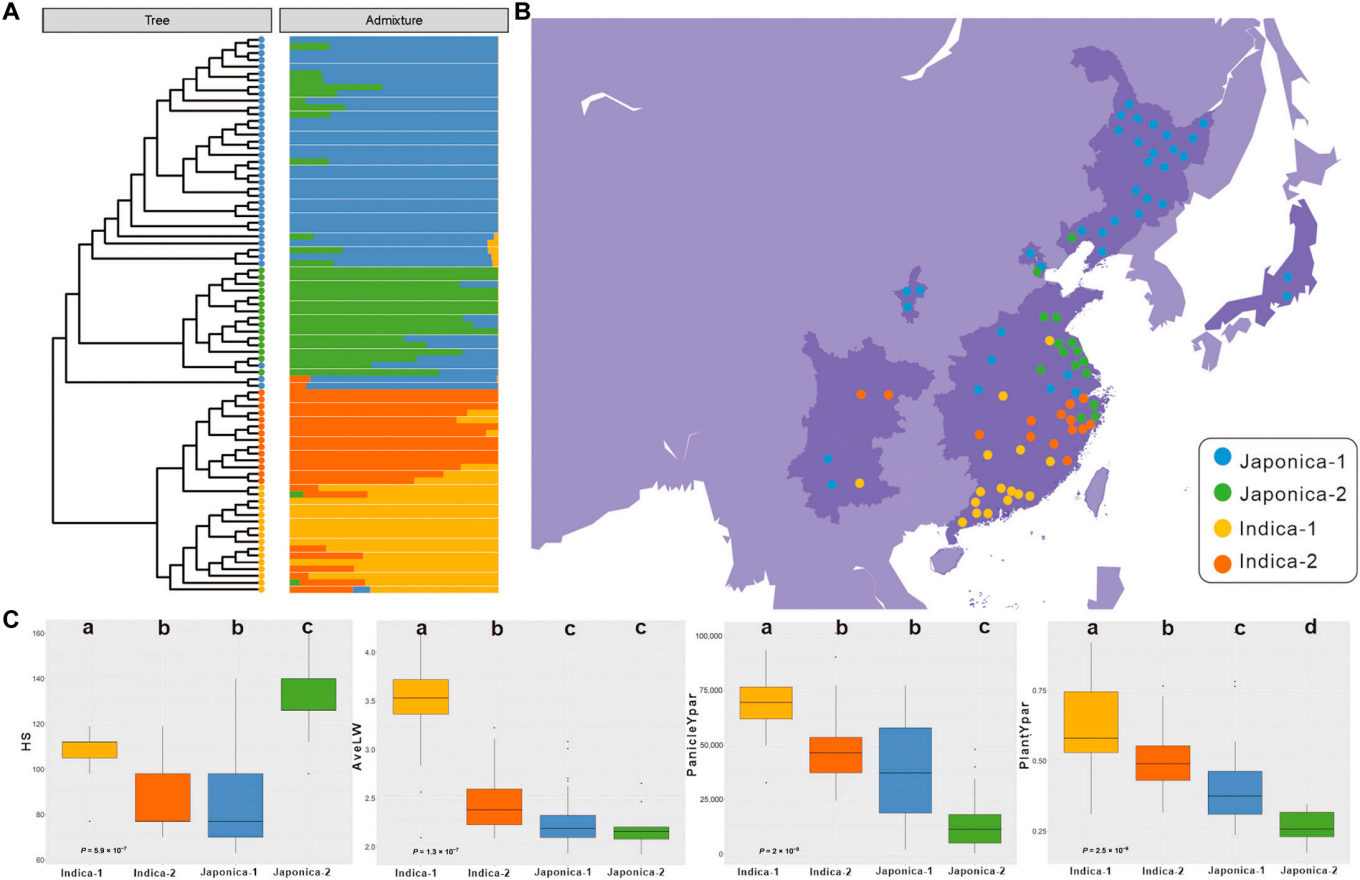
Division of the breeding regions and distribution of phenotypes in different rice genotypes. (A) Group clustering results of different rice genotypes. (B) Group divisions of different rice genotypes. (C) HS, AveLW, PanicleYPA, and PlantYpar values corresponding to different rice genotype groups.

The neighbor-joining tree and the division of the breeding region of all rice varieties are shown in Fig. 5A and B. Indica rice genotypes showed earlier senescence as a result of the Kruskal–Wallis test than the 2 japonica cultivar groups at the whole plant level and leaf level, as has been supported in previous studies [36]. HS (days to harvest from sowing), AveLW (mean value of the grain length/width radio), PanicleYPA (yellow projected area of panicles), and PlantYpar showed significant differences with the Kruskal–Wallis test within the 4 genotype groups (*P* < 0.05), while the pairwise comparison results among them differed. There were statistically significant differences between HS and PanicleYPA in different subspecies, but there were no significant differences between the Indica-2 group and the Japonica-1 group, potentially because of the similar genotypes of samples in these 2 groups (Fig. 5C). AveLW showed a significant difference between the indica and japonica subspecies groups, and Indica-1 grains were more elongated than the grains of the other 3 groups (Fig. 5C).

### GWAS with i-traits

**Fig. 6.**
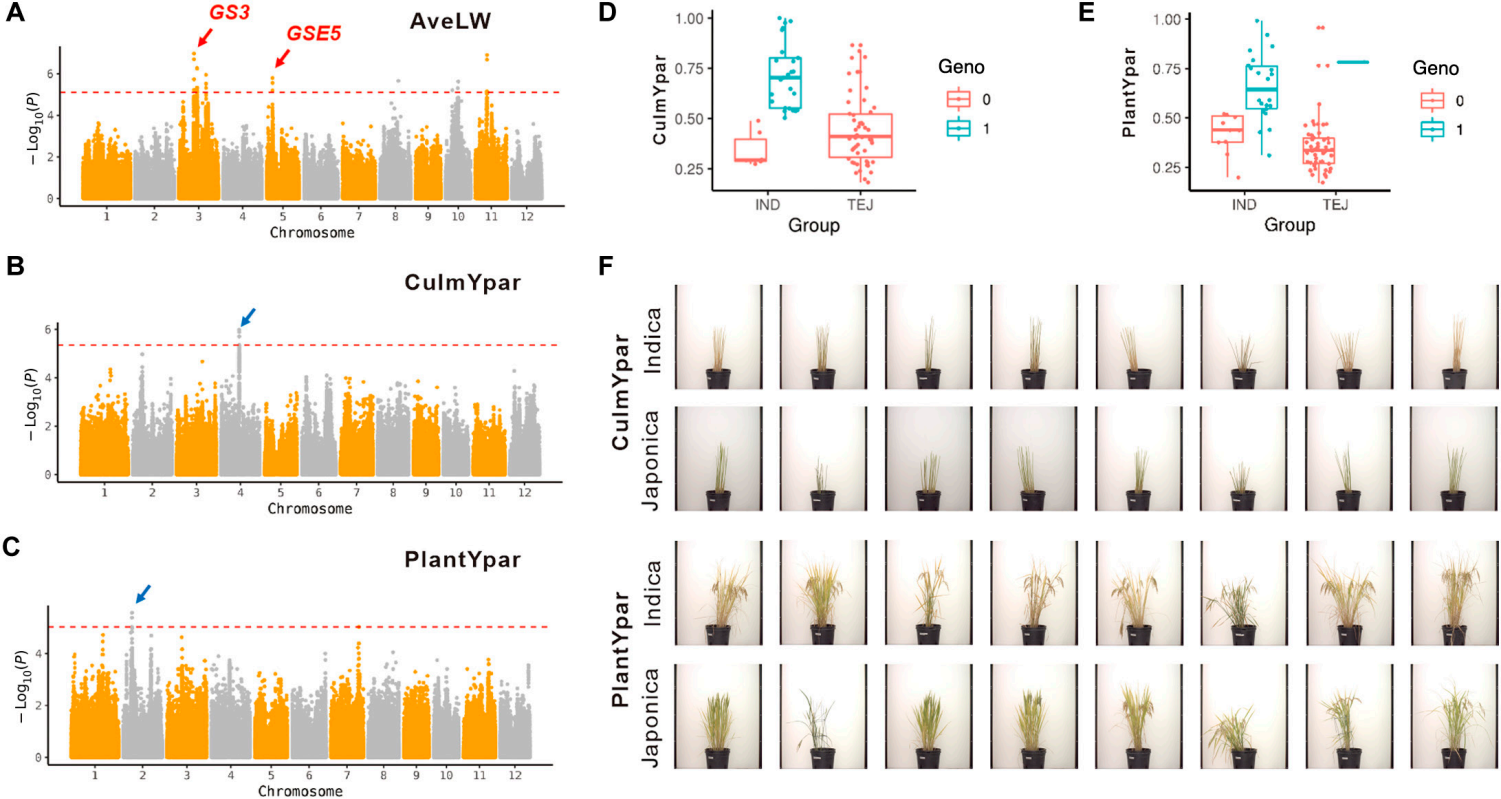
GWAS of traits. (A to C) Manhattan plots of AveLW, culmYpar, and PlantYpar. (D and E) Boxplots showing the phenotype distributions of the 2 genotypes on the lead SNP of culmYpar and PlantYpar. Accessions from the 2 major subspecies, indica (IND) and japonica (JAP), are displayed separately to exclude the impacts of the population structure. Genotype 0 indicates the reference genome (Nipponbare (NPB)) allele, and genotype 1 indicates an alternative allele. (F) Representative indica and japonica rice accessions exhibiting different culmYpar and PlantYpar values.

On the basis of these phenotypic data, we performed a GWAS with 87 accessions genotyped with 2,546,013 SNP markers. The significance threshold of the *P* value for each trait was determined by a permutation test (200 times; false discovery rate < 0.01). A total of 285 putative QTLs were detected for 33 traits (Table S22), including 255 for the raw phenotype traits and 30 for the organ–temporal dimension i-traits defined by the PCA. Although the small sample size limited the power and resolution of the association analysis, we were still able to detect some known quantitative trait loci, including *GSE5* [37] and *GS3* [38], which determined the rice grain shape (Fig. 6A). This suggests that we may be able to unearth novel genetic loci with relative confidence from this small population. We thus focused on some i-traits that are difficult to acquire by conventional methods but have strong biological implications.

The senescence of rice plants is accompanied by chlorophyll degradation and plant yellowing. The ratio of the yellow projected area provides a good indicator for measuring plant senescence. Two QTLs on chromosome 2 (Fig. 6B) and chromosome 4 (Fig. 6C) were detected for PlantYpar and culmYpar (ratio of the yellow projected area to the culmTPA), respectively. For both QTLs, the major allele in the indica subpopulation contributed to higher levels (Fig. 6D and E), which is consistent with the observation that indica rice exhibits earlier senescence in both whole plants and leaf organs than japonica rice [36]. These findings provide potential markers for further research on rice senescence and breeding.

**Fig. 7.**
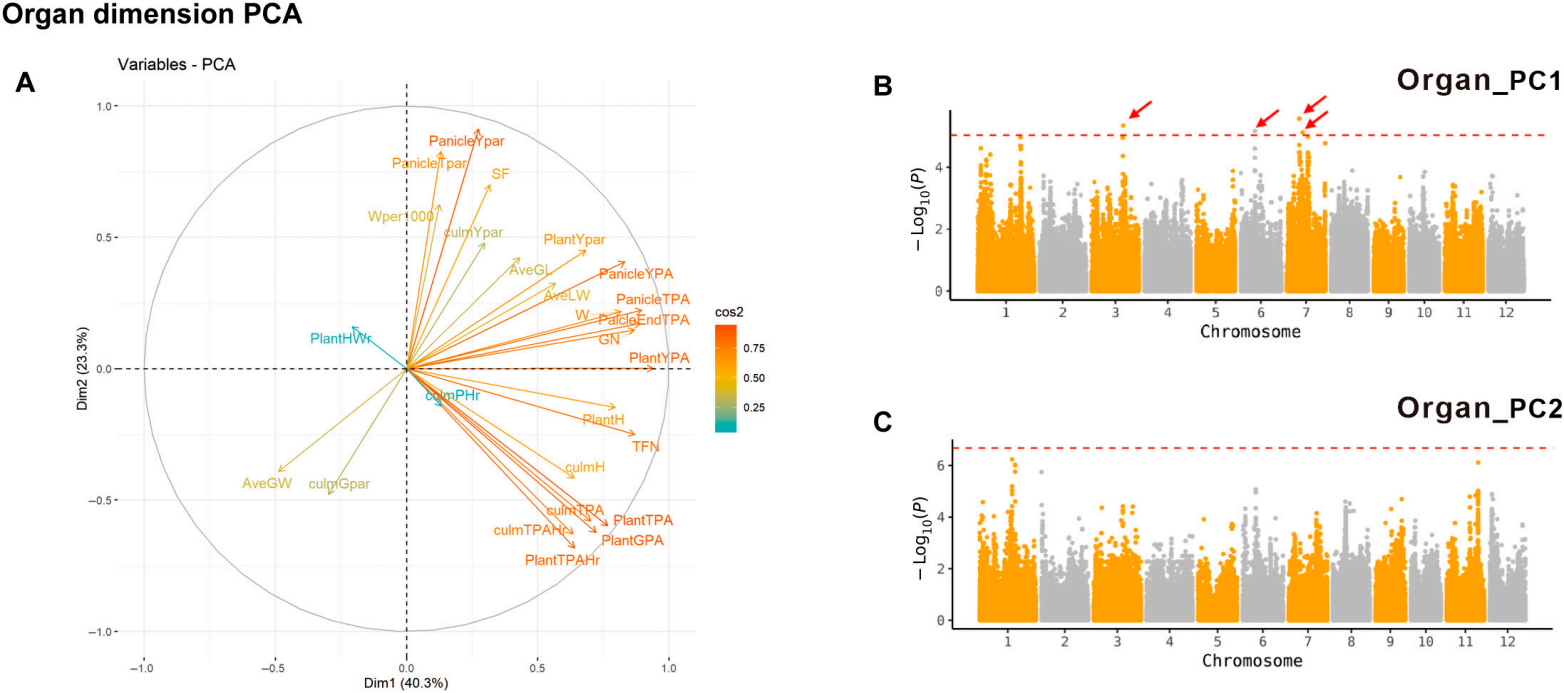
PCA and GWAS results with organ dimension traits. (A) Loading plot of Organ_PC1 and Organ_PC2. The proportions of variances in Organ_PC1 and Organ_PC12 are shown in parentheses. (B and C) GWAS results for Organ_PC1 (B) and Organ_PC2 (C) shown using the organ dimension traits. In the Manhattan plots, the horizontal dashed lines represent significant thresholds. The arrows indicate peaks that were further analyzed.

Performing GWAS on the PC values of raw traits may disclose genetic loci contributing to integrated variables that could be overlooked by using individual traits [39]. We then performed GWAS on the PCA results of organ- and time-related traits, and the specific details of the results are shown in Tables S8 and S9. For the Organ_PC traits, the first 2 PCs (Organ_PC1 and Organ_PC2) explained 40.3% and 23.3% of the total trait variance, respectively (Fig. 7A). Many traits related to plant and panicle architecture showed high positive loading on Organ_PC1, including the plant yellow projected area (PlantYPA), total spikelet number (TFN), plant height (PlantH), culmTPA, and PlantTPA. Organ_PC2 may be related to panicle growth and maturity. We detected 4 QTLs using Organ_PC1, all of which overlapped with the QTLs for raw traits such as the PlantTPA, culmTPA, and HS (Fig. 7B and C).

**Fig. 8.**
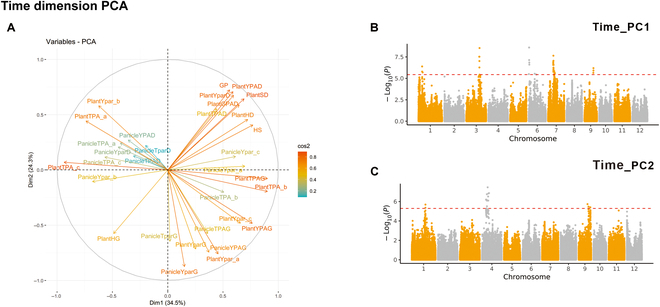
PCA and GWAS results with temporal dimension traits. (A) Loading plot for Time_PC1 and Time_PC2. The proportions of variances in Time_PC1 and Time_PC2 are shown in parentheses. (B and C) GWAS results for Time_PC1 (B) and Time_PC2 (C) shown using the temporal dimension traits. In the Manhattan plots, the horizontal dashed lines represent significant thresholds.

For the Time_PC traits, the first 2 PCs (Time_PC1 and Time_PC2) explained 34.5% and 24.3% of the total trait variance, respectively (Fig. 8A). Time_PC1 was highly related to the plant TPAG (daily growth value of the PlantTPA). Time_PC2 may have been related to the panicle growth and maturity rates. We detected 26 QTLs for the temporal PCA values, most of which overlapped with QTLs for the PlantTPAG, PlantHG (daily growth value of the height of the whole plant), and related parameters. Among them, a QTL on chromosome 3, with a lead SNP on chromosome 3 24262598, was detected by both the organ and temporal PCs, indicating that this locus deserves further study in the future involving confirmations using larger panels (Fig. 8B and C).

## Discussion

Confined to the limitations of traditional phenotyping involving the high labor cost, low efficiency, and high time consumption, most previous GWAS studies have usually focused on traditional phenotypes obtained manually at a specific point in time, generally at the mature stage, with characteristics of relative simplicity and the serious difficulty of large-number sampling; thus, with previous methods, it is almost impossible to perform vertical analyses in the temporal dimension. The growth of rice is a dynamic process, and the acquisition of phenotypes at the end time point is greatly affected by human activities, which are frequently obtained at the expense of integrity with destructive methods. With the development of high-throughput phenotype facilities, phenotype traits can be obtained precisely by ensuring high-throughput and nondestructive high-density time points that provide different research directions. In this study, we developed a strategy for the acquisition and analysis of image-based phenomes from the individual to organ level in rice during the whole growth period.

On the basis of the i-trait phenomes obtained here, up to 84.8% of the phenotypic variance in the rice yield could be explained by 8 traits among all i-traits considered, and this traits could thus be applied to predict the final rice yield better than predictions performed with other trait groups. This finding indicates that the i-traits obtained here can comprehensively reflect the growth status of rice, with the exception of the final yield.

To further explore the good environmental adaptability of the crop growth and development model, which would also show high inosculation with the latitude of the breeding region, the breeding region was divided into different latitude levels, and the correlations between all the image-based phenotypic traits of the elite rice cultivars and the regional latitude are shown in Fig. 4. Interestingly, in addition to the HS of japonica rice showing an increasing negative correlation with the region latitude, some traits showed correlations with the regional latitude with significant differences among rice accession groups, such as PanicleTPA and TFN of indica rice, which may explain the difference in the yields of different rice cultivars with the difference in the geographical environment of the breeding region. Moreover, the differences in rice subspecies were analyzed using the image-based phenotypic traits, which, in addition to PlantYpar, indicated the senescence degree at the whole-plant and leaf level, and the HS, AveLW, and PanicleYPA terms also showed significant differences.

Combining the high-throughput rice phenotyping platform and GWAS to unlock the genetic information coded in rice, more than 255 putative QTLs for i-traits were identified, and the known identification of GSE5 and GS3 also most likely eliminated the limitation of the power of association analysis with a small population. The life span of rice showed a close relationship with plant senescence, which is also a basic problem in evolutionary ecology. PlantYpar was defined as the value of the ratio of the yellow projection area to the overall color distribution of the whole plant, regarded as an important quantitative index of plant senescence, and the detection of QTLs was thus of great significance. The indica rice displayed earlier senescence than japonica rice [36], and the identification of the QTLs of PlantYpar and culmYpar on chromosome 2 and chromosome 4 offered potential markers for studying rice yield maximization.

Furthermore, PCA was also performed for the characteristics of the rice accessions in the organ–temporal dimension, and GWAS using PC scores was utilized to identify genetic factors. For organ dimension traits, Organ_PC1 captured 40.3% of the whole-trait variations in the organ dimension, whereas Organ_PC2 captured 23.3% of the variations in which Organ_PC1 primarily affected plant architecture and Organ_PC2 may have been related to panicle growth and maturity. Comparing the temporal dimension traits, Time_PC1 and Time_PC2 captured 34.5% and 24.3% of the total traits in the temporal dimension, respectively, which may have been related to the heading date and panicle growth-related traits. Among the PC values of the organ and temporal traits, a QTL on chromosome 3, with a lead SNP on chromosome 3: 24262598, was detected by both the organ and temporal PCs, indicating that this locus is deserving of further studies in the future involving confirmations with larger panels. Note that performing GWAS on the traits integrated by PCA is a double-edged sword, as it may not only help resolve the potential loci associated with complex traits but also cause more serious confounding effects, especially when using panels with strong population structures and traits with complex genetic control factors. As a proof of concept, this study demonstrates the potential value of this pathway in high-throughput phenomics studies and provides a reference for further similar studies in the future.

### Conclusion

In summary, in this work, on the basis of a high-throughput phenotyping tool, we extracted phenotypic traits by further analyzing the traits obtained across the whole growth stages of rice from the individual level to the organ level and identified trait-related QTLs with GWAS, thus providing a new research direction for heterosis breeding.

## Data Availability

The related data and code supporting the conclusion for this article will be made available on the following website: http://plantphenomics.hzau.edu.cn/usercrop/Rice/download.

## Supplementary Materials

Supplementary 1 Movie S1. Dynamic graph of the processing method. Note S1. Trait analysis technical documentation. Table S1. Information on *Oryza sativa*. Table S2. Statistical summary of the 6 developed models for estimating the panicle dry weight. Table S3. Summary of the 5-fold cross-validation results of model 5 for estimating the panicle dry weight. Table S4. Statistical summary of the 6 developed models for estimating the whole-plant dry weight. Table S5. Summary of the 5-fold cross-validation results of model 5 for estimating the whole-plant dry weight. Table S6. Statistical summary of the 6 developed models for estimating the culm dry weight. Table S7. Summary of the 5-fold cross-validation of model 5 for estimating the dry culm weight. Table S8. Summary of the specific indexes associated with the first 2 PCs for organ dimension traits. Table S9. Summary of the specific indexes associated with the first 2 PCs for temporal dimension traits. Table S10. Evaluation results of the segmentation of 10 randomly selected images. Table S11. Model fitting results of PlantTPA. Table S12. Model fitting results of PlantYpar. Table S13. Model fitting results of PanicleTPA. Table S14. Model fitting results of PanicleYpar. Table S15. Statistical details of the coefficients of the selected model with plant-related traits for estimating yield. Table S16. Statistical details of the coefficients of the selected model with panicle-related traits for estimating yield. Table S17. Statistical details of the coefficients of the selected model with culm-related traits for estimating yield. Table S18. Statistical details of the coefficients of the selected model with panicle growth-related traits for estimating yield. Table S19. Statistical details of the coefficients of the selected model with plant growth-related traits for estimating yield. Table S20. Statistical details of the coefficients of the selected model with all i-traits for estimating yield. Table S21. Trait groups of factors in the Mantel test analysis undertaken on the grain-related traits. Table S22. QTLs detected by GWAS. Fig. S1. The experimental arrangement of the rice material. Fig. S2. Variation in phenological traits. Fig. S3. Performance evaluation of the panicle segmentation of 3 randomly selected rice varieties. Fig. S4. Curve-fitting results of the temporal–organ developmental traits.
