# Supplementary material for: A Strategy for the Acquisition and Analysis of Image-Based Phenome in Rice during the Whole Growth Period
Source: Plant Phenomics. 2023 Jun 8;5:0058. doi: 10.34133/plantphenomics.0058 (PMC10249964; doi:10.34133/plantphenomics.0058)

**Traits Analysis Technical Documentation**

- **Biomass modeling and growth-related trait extraction**

Statistical analyses of the 6 models (including linear, quadratic, exponential, power, Gaussian, and triangular sine models) were performed with LabVIEW 2015 (National Instruments, USA) and MATLAB 2018b (MathWorks, USA)[1].

**
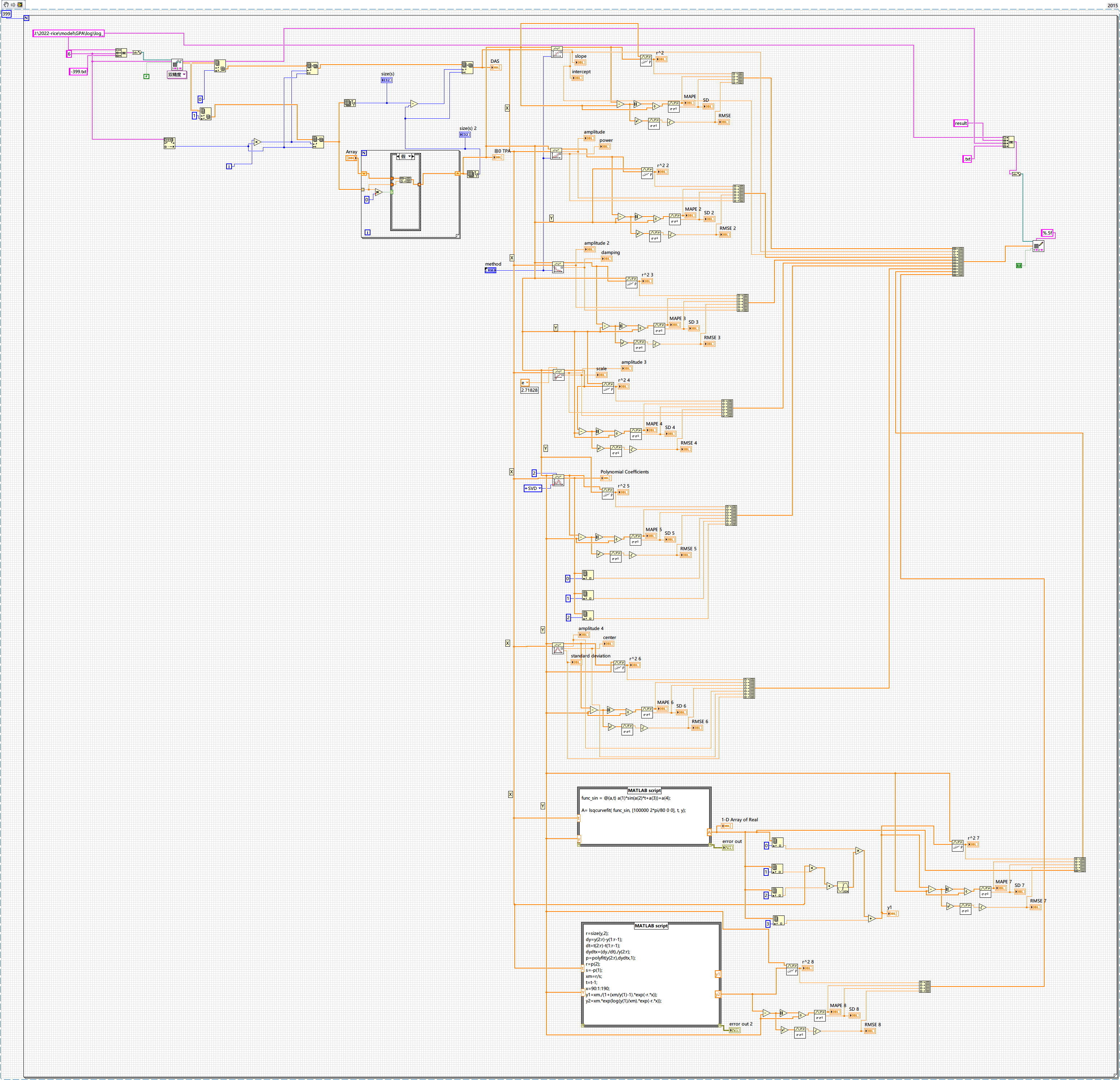
**

**References**

1. Li, H.T., et al., High-throughput phenotyping accelerates the dissection of the dynamic genetic architecture of plant growth and yield improvement in rapeseed. Plant Biotechnology Journal, 2020. 18(11): p. 2345-2353.

- **Performance evaluation of the phenotyping platform**

The performance evaluation of the phenotyping platform based on 6 models (including linear, quadratic, exponential, power, Gaussian, and triangular sine models) were performed with Python 3.8.


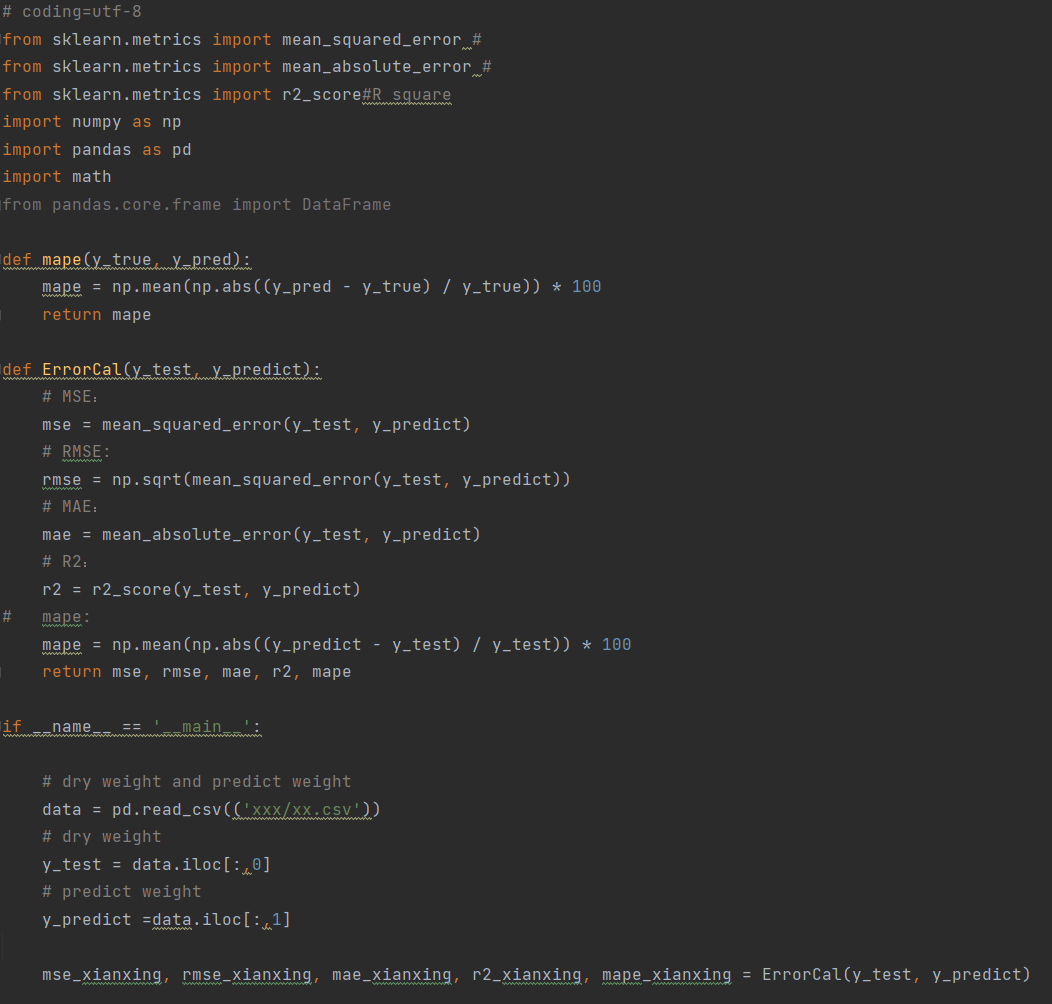


Grain yield prediction using phenotypic traits

1. Data import


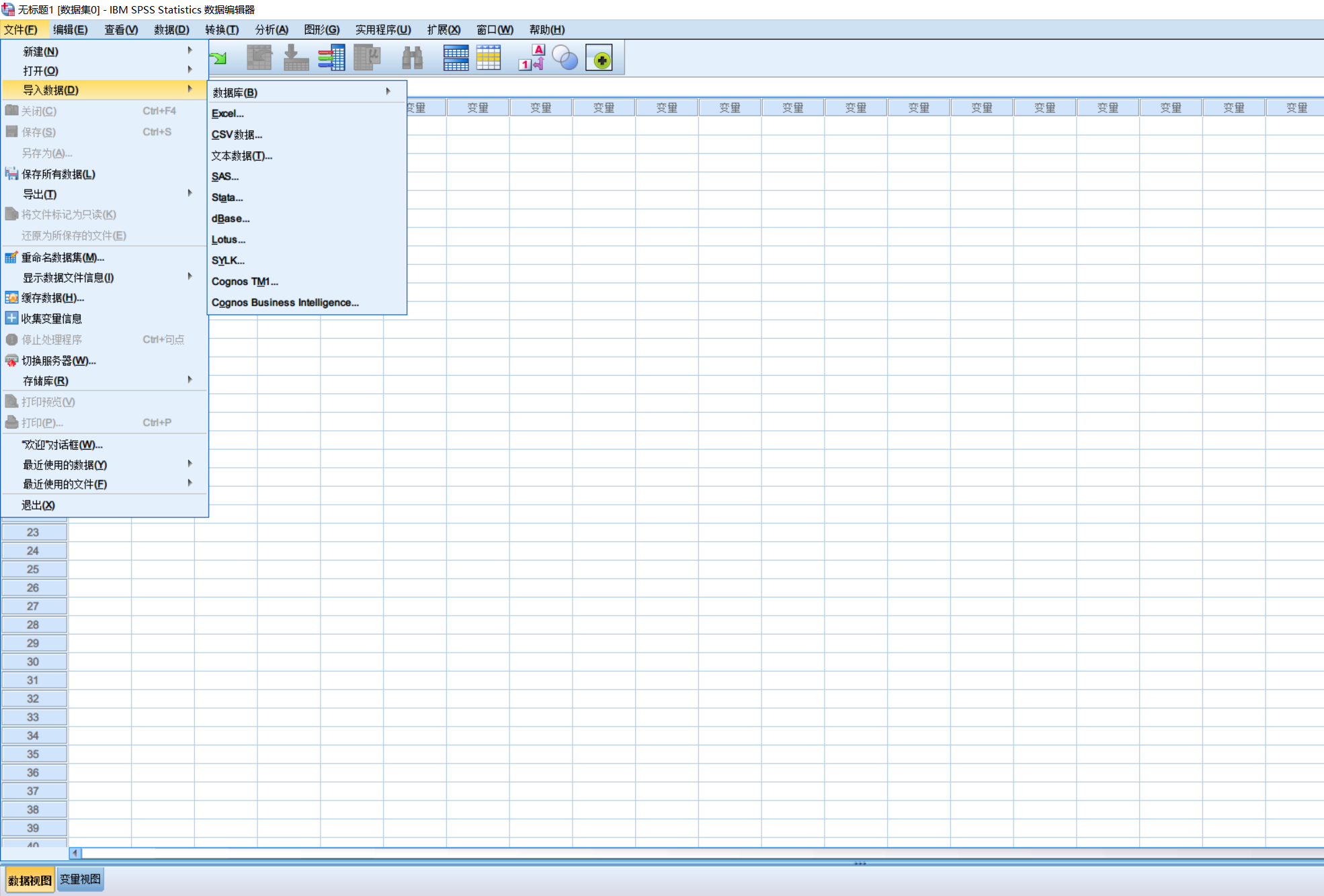


1. Selecting the function


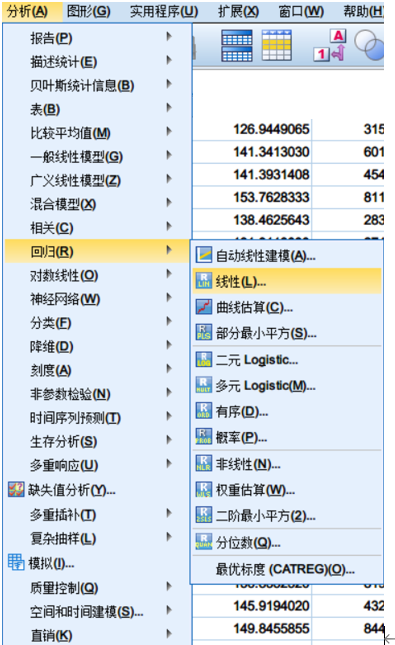


1. Setting 0.05 and 0.1 as the entry and removal values of the use probability of F, respectively.


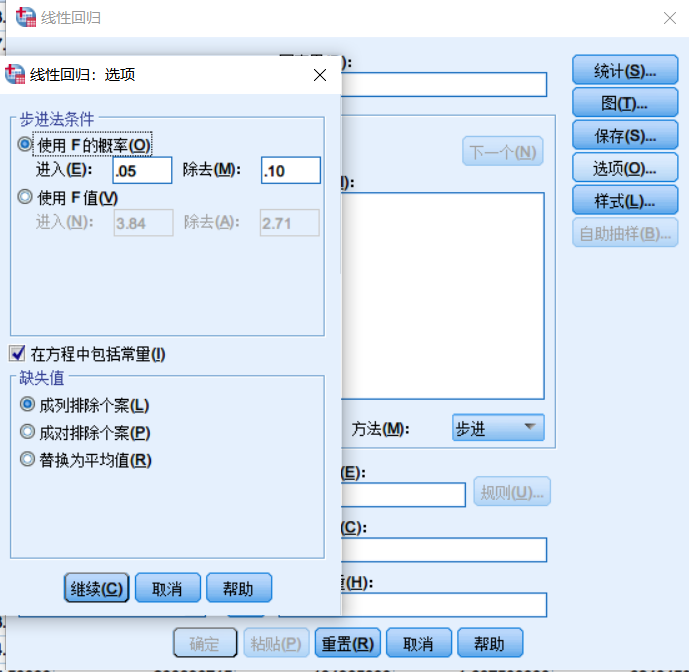


- **Kruskal–Wallis test and pairwise multiple comparisons**

The Kruskal‒Wallis test and Wilcoxon test were implemented on the ggsignif R software package (R Foundation for Statistical Computing, Austria).


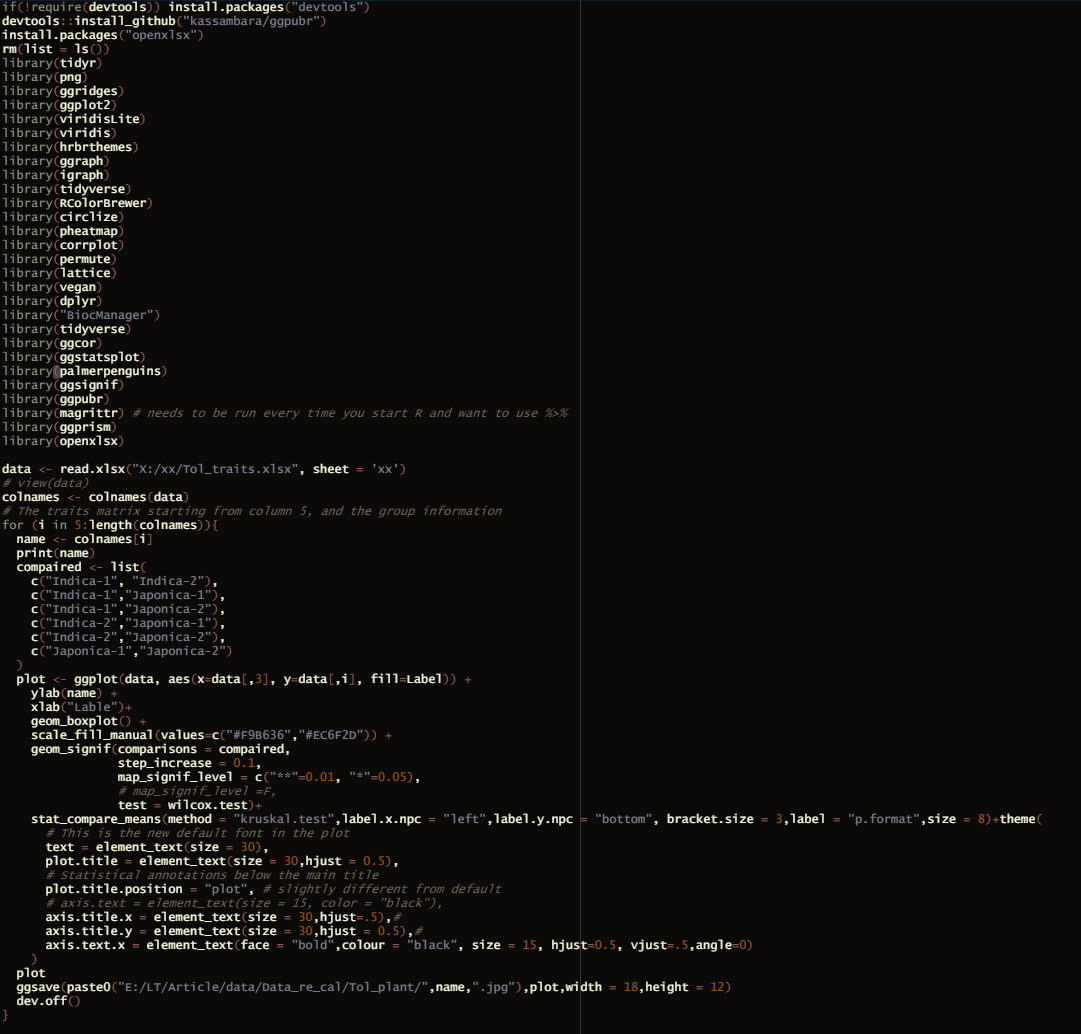


- **Principal component analysis**

PCA was performed using the correlation matrix of trait values in the pca R software packages (R Foundation for Statistical Computing, Austria) by setting “scale = TRUE”.


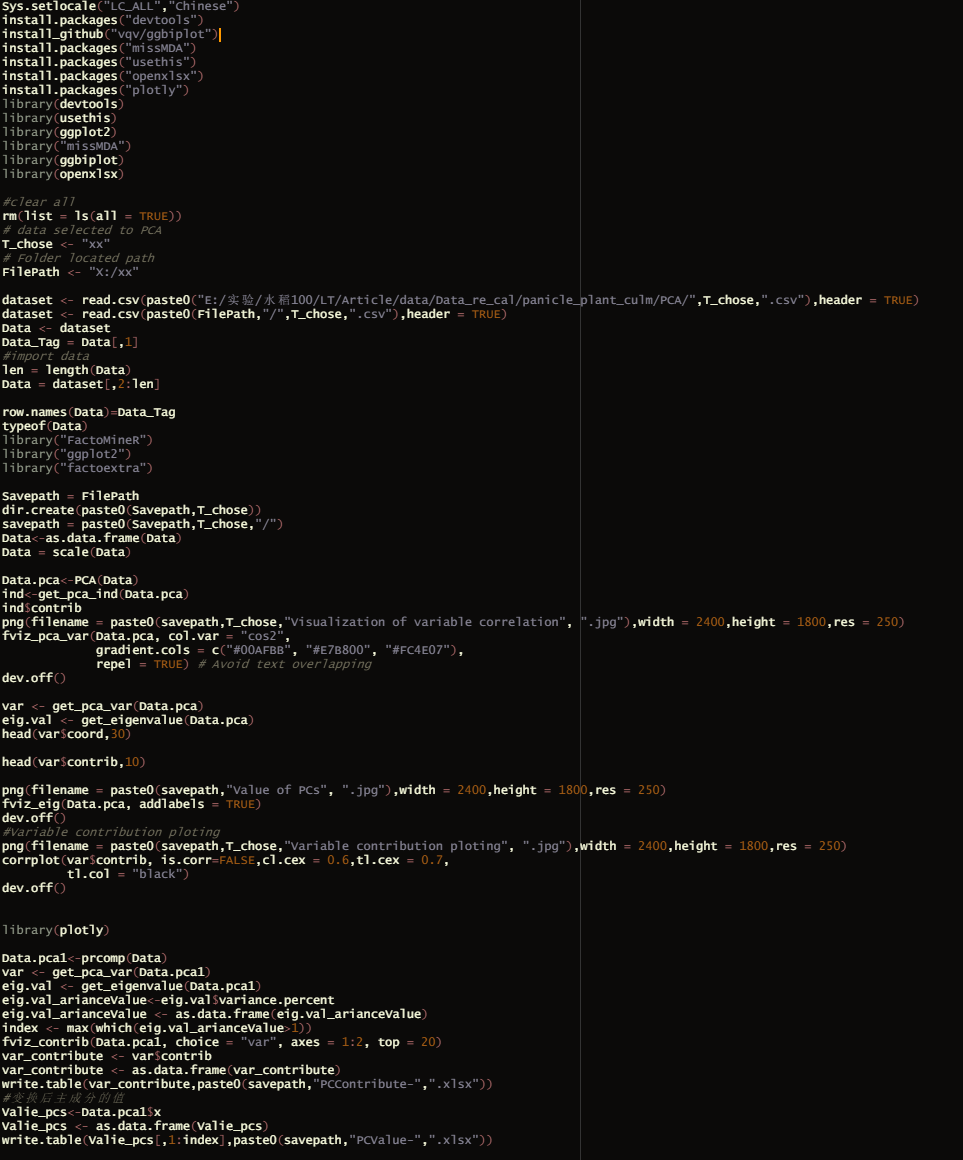

Supplement: Supplementary 1 — Movie S1. Dynamic graph of the processing method. Note S1. Trait analysis technical documentation. Table S1. Information on Oryza sativa. Table S2. Statistical summary of the 6 developed models for estimating the panicle dry weight. Table S3. Summary of the 5-fold cross-validation results of model 5 for estimating the panicle dry weight. Table S4. Statistical summary of the 6 developed models for estimating the whole-plant dry weight. Table S5. Summary of the 5-fold cross-validation results of model 5 for estimating the whole-plant dry weight. Table S6. Statistical summary of the 6 developed models for estimating the culm dry weight. Table S7. Summary of the 5-fold cross-validation of model 5 for estimating the dry culm weight. Table S8. Summary of the specific indexes associated with the first 2 PCs for organ dimension traits. Table S9. Summary of the specific indexes associated with the first 2 PCs for temporal dimension traits. Table S10. Evaluation results of the segmentation of 10 randomly selected images. Table S11. Model fitting results of PlantTPA. Table S12. Model fitting results of PlantYpar. Table S13. Model fitting results of PanicleTPA. Table S14. Model fitting results of PanicleYpar. Table S15. Statistical details of the coefficients of the selected model with plant-related traits for estimating yield. Table S16. Statistical details of the coefficients of the selected model with panicle-related traits for estimating yield. Table S17. Statistical details of the coefficients of the selected model with culm-related traits for estimating yield. Table S18. Statistical details of the coefficients of the selected model with panicle growth-related traits for estimating yield. Table S19. Statistical details of the coefficients of the selected model with plant growth-related traits for estimating yield. Table S20. Statistical details of the coefficients of the selected model with all i-traits for estimating yield. Table S21. Trait groups of factors in the Mantel [file plantphenomics.0058.f1.zip › Supplementary Note.docx]
