# Supplementary material for: A Strategy for the Acquisition and Analysis of Image-Based Phenome in Rice during the Whole Growth Period
Source: Plant Phenomics. 2023 Jun 8;5:0058. doi: 10.34133/plantphenomics.0058 (PMC10249964; doi:10.34133/plantphenomics.0058)
Supplement: Supplementary 1 — Movie S1. Dynamic graph of the processing method. Note S1. Trait analysis technical documentation. Table S1. Information on Oryza sativa. Table S2. Statistical summary of the 6 developed models for estimating the panicle dry weight. Table S3. Summary of the 5-fold cross-validation results of model 5 for estimating the panicle dry weight. Table S4. Statistical summary of the 6 developed models for estimating the whole-plant dry weight. Table S5. Summary of the 5-fold cross-validation results of model 5 for estimating the whole-plant dry weight. Table S6. Statistical summary of the 6 developed models for estimating the culm dry weight. Table S7. Summary of the 5-fold cross-validation of model 5 for estimating the dry culm weight. Table S8. Summary of the specific indexes associated with the first 2 PCs for organ dimension traits. Table S9. Summary of the specific indexes associated with the first 2 PCs for temporal dimension traits. Table S10. Evaluation results of the segmentation of 10 randomly selected images. Table S11. Model fitting results of PlantTPA. Table S12. Model fitting results of PlantYpar. Table S13. Model fitting results of PanicleTPA. Table S14. Model fitting results of PanicleYpar. Table S15. Statistical details of the coefficients of the selected model with plant-related traits for estimating yield. Table S16. Statistical details of the coefficients of the selected model with panicle-related traits for estimating yield. Table S17. Statistical details of the coefficients of the selected model with culm-related traits for estimating yield. Table S18. Statistical details of the coefficients of the selected model with panicle growth-related traits for estimating yield. Table S19. Statistical details of the coefficients of the selected model with plant growth-related traits for estimating yield. Table S20. Statistical details of the coefficients of the selected model with all i-traits for estimating yield. Table S21. Trait groups of factors in the Mantel [file plantphenomics.0058.f1.zip › Supplementary Table 1-22.docx]

**Supplementary Table 1** **Information on Oryza sativa**

| Number | Oryza sativa | rice subspecies | Breeding area |
| --- | --- | --- | --- |
| 1 | Longjing31 | Japonica | Heilongjiang |
| 2 | Zhongjiazao17 | Indica | Zhejiang |
| 3 | Suijing18 | Japonica | Heilongjiang |
| 4 | Huanghuazhan | Indica | Guangdong |
| 5 | Nanjing9108 | Japonica | Jiangsu |
| 6 | Huaidao5 | Japonica | Jiangsu |
| 7 | Longjing46 | Japonica | Heilongjiang |
| 8 | Zhongzao39 | Indica | Zhejiang |
| 9 | Longjing39 | Japonica | Heilongjiang |
| 10 | Yanfeng47 | Japonica | Liaoning |
| 11 | Longjing43 | Japonica | Heilongjiang |
| 12 | Kendao12 | Japonica | Heilongjiang |
| 13 | Longjing26 | Japonica | Heilongjiang |
| 14 | Ningjing4 | Japonica | Jiangsu |
| 15 | NJ5055 | Japonica | Jiangsu |
| 16 | Lianjing7 | Japonica | Jiangsu |
| 17 | Longjing29 | Japonica | Heilongjiang |
| 18 | Xiushui134 | Japonica | Zhejiang |
| 19 | Wuyoudao4 | Japonica | Heilongjiang |
| 20 | Wuyunjing23 | Japonica | Jiangsu |
| 21 | Longdao 18 | Japonica | Heilongjiang |
| 22 | Wushansimiao | Indica | Guangdong |
| 23 | Jijing88 | Japonica | Jilin |
| 24 | Chujing28 | Japonica | Yunnan |
| Number | Oryza sativa | rice subspecies | Breeding area |
| 25 | Meixiangzhan-2 | Indica | Guangdong |
| 26 | Jia58 | Japonica | Zhejiang |
| 27 | Suxiu867 | Japonica | Jiangsu |
| 28 | Wankennuo1 | Japonica | Anhui |
| 29 | Kongyu131 | Japonica | Japan |
| 30 | Longjing21 | Japonica | Heilongjiang |
| 31 | Zhongzao33 | Indica | Zhejiang |
| 32 | Erzao18 | Indica | Hubei |
| 33 | WD68 | Japonica | Anhui |
| 34 | Fenghuazhan | Indica | Guangdong |
| 35 | Zhongzao 35 | Indica | Jiangxi |
| 36 | Huahang31 | Indica | Guangdong |
| 37 | Baijing1 | Japonica | Jilin |
| 38 | Wuyunjing21 | Japonica | Jiangsu |
| 39 | Yuejingsimiao2 | Indica | Guangdong |
| 40 | Jiafuzhan | Indica | Fujian |
| 41 | Changbai9 | Japonica | Jilin |
| 42 | Jiaxing8 | Indica | Zhejiang |
| 43 | LiaoXing1 | Japonica | Liaoning |
| 44 | Wuyunjiing7 | Japonica | Jiangsu |
| 45 | Guinongzhan | Indica | Guangdong |
| 46 | Jiayu948 | Indica | Zhejiang |
| 47 | Texianzhan25 | Indica | Guangdong |
| 48 | dangyujing10 | Japonica | Anhui |
| 49 | Songjing9 | Japonica | Heilongjiang |
| Number | Oryza sativa | rice subspecies | Breeding area |
| 50 | Longjing47 | Japonica | Heilongjiang |
| 51 | Qilisimiao | Indica | Guangdong |
| 52 | jinzao47 | Indica | Zhejiang |
| 53 | Yangdao6 | Indica | Jiangsu |
| 54 | Jiahua1 | Japonica | Zhejiang |
| 55 | Runnong11 | Japonica | Shandong |
| 56 | Zhefu802 | Indica | Zhejiang |
| 57 | Wuyujing3 | Japonica | Jiangsu |
| 58 | Guichao2 | Indica | Guangdong |
| 59 | Eyi105 | Japonica | Hubei |
| 60 | Qiuguang | Japonica | Japan |
| 61 | Xiangzaoxian7 | Indica | Hunan |
| 62 | Ganzaoxian7 | Indica | Jiangxi |
| 63 | Yujing6 | Japonica | Henan |
| 64 | Hong410 | Indica | Fujian |
| 65 | Xiangaizao9 | Indica | Hunan |
| 66 | 78130 | Indica | Fujian |
| 67 | Xiushui11 | Japonica | Zhejiang |
| 68 | Xiangzaoxian1 | Indica | Hunan |
| 69 | Ganwanxian30 | Indica | Jiangxi |
| 70 | Huhongzao1 | Indica | Sichuan |
| 71 | Liaojing9 | Japonica | Liaoning |
| 72 | Jingyue1 | Japonica | Beijing |
| 73 | Tong35 | Japonica | Jilin |
| 74 | Ningjing16 | Japonica | Ningxia |
| Number | Oryza sativa | rice subspecies | Breeding area |
| 75 | Huke3 | Indica | Sichuan |
| 76 | XD68-11 | Japonica | Henan |
| 77 | Qiutianxiaoting | Japonica | Jilin |
| 78 | XD18 | Japonica | Henan |
| 79 | Jinyuan45 | Japonica | Tianjin |
| 80 | Hushuang101 | Japonica | Shanghai |
| 81 | Yunjing26 | Japonica | Yunnan |
| 82 | Ningjing41 | Japonica | Ningxia |
| 83 | Jinhe1 | Japonica | Liaoning |
| 84 | Shennong315 | Japonica | Liaoning |
| 85 | Shengdao14 | Japonica | Shandong |
| 86 | Heijing10 | Japonica | Heilongjiang |
| 87 | Jinyuan85 | Japonica | Tianjin |
| 88 | Hongyou6 | Indica | Yunnan |
| 89 | Ningjing57 | Japonica | Ningxia |
| 90 | Huangzhan | Indica | Guizhou |
| 91 | Baikehanhe | Japonica | Guizhou |
| 92 | Aizihuangkenuo | Japonica | Guizhou |
| 93 | Qingzhong | Japonica | Shanghai |

**Supplementary Table 2 Statistical summary of the 6 developed models for estimating the panicle dry weight**

| **Traits** | **NO.** | **Model classification** | **Model solution** | **coefficient** | **Result of forecast model evaluation** | | | |
| --- | --- | --- | --- | --- | --- | --- | --- | --- |
|  |  |  |  |  | **MAPE** | **RMSE(g)** | **MAE**  **(g)** | **R^2^** |
| DryWeightOfPanicle | 1 | Linear model | DWP = ax + b | a = 4.400E-04 | 31.46% | 9.621 | 7.459 | 0.657 |
|  |  |  |  | b = 4.756E+00 |  |  |  |  |
|  | 2 | Power model | DWP = ax^b^ | a = 5.322E-03 | 32.81% | 9.336 | 7.339 | 0.677 |
|  |  |  |  | b = 7.913E-01 |  |  |  |  |
|  | 3 | Exponential model | DWP = ae^bx^ | a = 1.664E+01 | 48.63% | 11.411 | 9.504 | 0.517 |
|  |  |  |  | b = 9.670E-06 |  |  |  |  |
|  | 4 | Logarithm model | DWP = aln(bx) | a = 1.913E+01 | 44.54% | 10.430 | 8.204 | 0.597 |
|  |  |  |  | b = 9.885E-05 |  |  |  |  |
|  | 5 | Quadratic model | DWP = ax^2^ + bx +c | a = -3.000E-09 | 28.76% | 8.667 | 6.382 | 0.721 |
|  |  |  |  | b = 8.626E-04 |  |  |  |  |
|  |  |  |  | c = -6.717E+00 |  |  |  |  |
|  | 6 | Gussian model | $\mathrm{DWP}=ae^{{-(x-b)}^{2}/2c^{2}}$ | a = 5.682E+01 | 23.70% | 9.570 | 6.650 | 0.660 |
|  |  |  |  | b = 1.086E+05 |  |  |  |  |
|  |  |  |  | c = 4.511E+04 |  |  |  |  |

**Supplementary Table 3 Summary of the 5-fold cross-validation results of Model 5 for estimating the panicle dry weight**

| **Traits** | **No.** | **MAPE** | **RMSE(g)** | **MAE(g)** | **R^2^** |
| --- | --- | --- | --- | --- | --- |
| DryWeightOfPanicle | 1 | 28.62% | 11.656 | 9.013 | 0.617 |
|  | 2 | 19.18% | 5.252 | 3.762 | 0.905 |
|  | 3 | 30.66% | 7.005 | 5.962 | 0.768 |
|  | 4 | 32.89% | 8.929 | 6.066 | 0.693 |
|  | 5 | 35.66% | 10.097 | 8.005 | 0.429 |
|  | Mean | 29.40% | 8.588 | 6.562 | 0.682 |
|  | SD | 0.056 | 2.256 | 1.819 | 0.158 |

**Supplementary Table 4 Statistical summary of the 6 developed models for estimating the whole-plant dry weight**

| **Traits** | **NO.** | **Model classification** | **Model solution** | **coefficient** | **Result of forecast model evaluation** | | | | |
| --- | --- | --- | --- | --- | --- | --- | --- | --- | --- |
|  |  |  |  |  | **MAPE** | | **RMSE(g)** | **MAE(g)** | **R^2^** |
| DryWeightOfWholeplant | 1 | Linear model | DWPlant = ax + b | a = 2.145E-04 | 14.62% | 13.054 | | 9.083 | 0.837 |
|  |  |  |  | b = 1.523E+00 |  |  | |  |  |
|  | 2 | Power model | DWPlant = ax^b^ | a = 5.171E-04 | 15.43% | 12.968 | | 9.174 | 0.840 |
|  |  |  |  | b = 9.333E-01 |  |  | |  |  |
|  | 3 | Exponential model | DWPlant = ae^bx^ | a = 2.717E+01 | 23.86% | 15.687 | | 12.369 | 0.765 |
|  |  |  |  | b = 2.784E-06 |  |  | |  |  |
|  | 4 | Logarithm model | DWPlant = aln(bx) | a = 2.784E-06 | 17.75% | 12.984 | | 9.192 | 0.839 |
|  |  |  |  | b = 1.198E-05 |  |  | |  |  |
|  | 5 | Quadratic model | DWPlant = ax^2^ + bx +c | a = -3.000E-10 | 14.45% | 12.152 | | 8.203 | 0.859 |
|  |  |  |  | b = 4.080E-04 |  |  | |  |  |
|  |  |  |  | c = -2.299E+01 |  |  | |  |  |
|  | 6 | Gussian model | $\mathrm{DWPlant}=ae^{{-(x-b)}^{2}/2c^{2}}$ | a = 1.097E+02 | 20.88% | 14.041 | | 10.997 | 0.812 |
|  |  |  |  | b = 5.010E+05 |  |  | |  |  |
|  |  |  |  | c = 1.827E+05 |  |  | |  |  |

**Supplementary Table 5 Summary of the 5-fold cross-validation results of Model 5 for estimating the whole-plant dry weight**

| **Traits** | **No.** | **MAPE** | **RMSE(g)** | **MAE(g)** | **R^2^** |
| --- | --- | --- | --- | --- | --- |
| DryWeightOfWholeplant | 1 | 16.07% | 17.912 | 11.081 | 0.764 |
|  | 2 | 10.67% | 8.145 | 6.777 | 0.907 |
|  | 3 | 23.58% | 10.738 | 8.945 | 0.911 |
|  | 4 | 25.95% | 18.149 | 13.664 | 0.625 |
|  | 5 | 11.93% | 11.032 | 8.682 | 0.808 |
|  | Mean | 17.64% | 13.195 | 9.830 | 0.803 |
|  | SD | 0.061 | 4.074 | 2.353 | 0.106 |

**Supplementary Table 6 Statistical summary of the 6 developed models for estimating the culm dry weight**

| **Traits** | **NO.** | Model classification | Model solution | coefficient | **Result of forecast model evaluation** | | | | |
| --- | --- | --- | --- | --- | --- | --- | --- | --- | --- |
|  |  |  |  |  | **MAPE** | **RMSE(g)** | | **MAE(g)** | **R^2^** |
| DryWeightOfCulm | 1 | Linear model | DWC = ax + b | a = 3.532E-04 | 32.04% | | 12.925 | 9.513 | 0.545 |
|  |  |  |  | b = 3.391E-02 |  | |  |  |  |
|  | 2 | Power model | DWC = ax^b^ | a = 1.320E-03 | 37.01% | | 12.814 | 10.025 | 0.552 |
|  |  |  |  | b = 8.870E-01 |  | |  |  |  |
|  | 3 | Exponential model | DWC = ae^bx^ | a = 1.675E+01 | 52.78% | | 14.866 | 12.190 | 0.398 |
|  |  |  |  | b = 6.966E-06 |  | |  |  |  |
|  | 4 | Logarithm model | DWC = aln(bx) | a = 3.146E+01 | 33.30% | | 11.897 | 9.374 | 0.614 |
|  |  |  |  | b = 3.395E-05 |  | |  |  |  |
|  | 5 | Quadratic model | DWC = ax^2^ + bx +c | a = -3.000E-09 | 33.42% | | 10.890 | 8.735 | 0.677 |
|  |  |  |  | b = 9.819E-04 |  | |  |  |  |
|  |  |  |  | c = -2.712E+01 |  | |  |  |  |
|  | 6 | Gussian model | $\mathrm{DW}C=ae^{{-(x-b)}^{2}/2c^{2}}$ | a = 5.770E+01 | 39.92% | | 12.302 | 9.758 | 0.588 |
|  |  |  |  | b = 1.318E+05 |  | |  |  |  |
|  |  |  |  | c = 3.723E+04 |  | |  |  |  |

**Supplementary Table 7 Summary of the 5-fold cross-validation of Model 5 for estimating the dry culm weight**

| **Traits** | **No.** | **MAPE** | **RMSE(g)** | **MAE(g)** | **R^2^** |
| --- | --- | --- | --- | --- | --- |
| DryWeightOfCulm | 1 | 27.41% | 10.299 | 8.430 | 0.709 |
|  | 2 | 30.35% | 12.951 | 10.418 | 0.590 |
|  | 3 | 42.65% | 9.973 | 8.309 | 0.724 |
|  | 4 | 44.10% | 11.915 | 9.449 | 0.512 |
|  | 5 | 29.23% | 10.535 | 8.317 | 0.647 |
|  | Mean | 34.75% | 11.135 | 8.985 | 0.636 |
|  | SD | 0.071 | 1.124 | 0.834 | 0.079 |

**Supplementary Table 8 Summary of the specific indexes associated with the first 2 PCs for organ-dimension traits**

| **Traits** | **Organ_PC1** | **Organ_PC2** |
| --- | --- | --- |
| PlantH | 6.0356301 | 0.362827511 |
| PlantTPA | 5.616144222 | 5.899779673 |
| PlantHWr | 0.410318598 | 0.421217962 |
| PlantTPAHr | 3.914234894 | 7.69460747 |
| PlantYpar | 4.455109991 | 3.361802382 |
| PlantYPA | 8.451405015 | 2.92E-06 |
| PlantGPA | 4.981606079 | 6.430670647 |
| PanicleTPA | 7.675102029 | 0.818559747 |
| PaicleEndTPA | 7.575134329 | 0.486517272 |
| PanicleTpar | 0.164010543 | 11.3002986 |
| PanicleYpar | 0.719279561 | 13.74493921 |
| PanicleYPA | 6.618550981 | 2.731001746 |
| culmH | 3.895972787 | 2.853184898 |
| culmTPA | 4.707600423 | 5.551572046 |
| culmTPAHr | 3.83708708 | 6.550298431 |
| culmGpar | 0.844259271 | 3.783987246 |
| culmYpar | 0.844259558 | 3.783986155 |
| culmPHr | 0.168359591 | 0.334857192 |
| AveGL | 1.780439281 | 2.936337952 |
| AveLW | 3.088923511 | 1.739607533 |
| AveGW | 2.264043328 | 2.525799457 |
| TFN | 7.230892454 | 1.035308958 |
| Wper1000 | 0.150848725 | 6.440477876 |
| W | 6.402659431 | 0.777491938 |
| **Traits** | **Organ_PC1** | **Organ_PC2** |
| SF | 0.961393395 | 8.079631411 |
| GN | 7.206734822 | 0.355233767 |
| **eigenvalue** | 1.05E+01 | 6.05E+00 |
| **percentage of variance** | 4.03E+01 | 2.33E+01 |
| **cumulative percentage of variance** | 40.2579 | 63.54002 |

**Supplementary Table 9 Summary of the specific indexes associated with the first 2 PCs for temporal-dimension traits**

| **Traits** | **Time_PC1** | **Time_PC2** |
| --- | --- | --- |
| GP | 3.95692549 | 5.670336461 |
| HS | 6.06574257 | 1.544273041 |
| PlantSD | 5.384985217 | 4.391061781 |
| PlantHD | 5.543055217 | 1.995651221 |
| PlantTPAD | 3.136378787 | 2.389349919 |
| PlantYparD | 4.125513118 | 5.06685283 |
| PlantYPAD | 4.30433857 | 5.335993821 |
| PlantGPAD | 5.120806861 | 3.153537324 |
| PanicleTPAD | 0.676888121 | 0.200876219 |
| PanicleTparD | 0.348152639 | 0.869432522 |
| PanicleYparD | 1.656341698 | 0.789548687 |
| PanicleYPAD | 0.903915062 | 1.072266104 |
| PanicleTPAG | 0.705748506 | 4.617780748 |
| PanicleTparG | 0.02573949 | 5.500435724 |
| PanicleYparG | 0.020328701 | 10.30348506 |
| PanicleYPAG | 0.690674872 | 7.795486933 |
| PanicleTPA_a | 1.387408214 | 0.96019341 |
| PanicleTPA_b | 2.092355967 | 0.762433213 |
| PanicleTPA_c | 2.824426949 | 0.37110545 |
| PanicleYpar_a | 4.653953269 | 0.026368509 |
| PanicleYpar_b | 4.590304409 | 0.006459109 |
| PanicleYpar_c | 3.863414846 | 0.021162584 |
| PlantHG | 3.033238089 | 3.515278708 |
| PlantTPAG | 6.770725028 | 0.236786978 |
| **Traits** | **PC1** | **PC2** |
| PlantYparG | 0.231112131 | 6.99687453 |
| PlantYPAG | 4.543668023 | 4.08425088 |
| PlantTPA_a | 3.306123674 | 3.304661075 |
| PlantTPA_b | 6.10219384 | 1.164997497 |
| PlantTPA_c | 7.08536461 | 0.435866666 |
| PlantYpar_a | 1.057211877 | 8.132681558 |
| PlantYpar_b | 2.604735307 | 5.319994284 |
| PlantYpar_c | 3.188228847 | 3.964517157 |
| **eigenvalue** | 1.10E+01 | 7.79E+00 |
| **percentage of variance** | 3.45E+01 | 2.43E+01 |
| **cumulative percentage of variance** | 34.51216 | 58.85939 |

**Supplementary Table 10 Evaluation results of the segmentation of 10 randomly selected images**

| **Tag** | **IoU** | **Precision** | **Recall** | **F-measure** |
| --- | --- | --- | --- | --- |
| 11 | 0.827787 | 0.864023 | 0.95178 | 0.905781 |
| 26 | 0.832449 | 0.917045 | 0.900239 | 0.908565 |
| 28 | 0.823434 | 0.881603 | 0.925815 | 0.903169 |
| 33 | 0.778791 | 0.826949 | 0.930425 | 0.875641 |
| 93 | 0.767357 | 0.872599 | 0.864175 | 0.868367 |
| 133 | 0.828597 | 0.863989 | 0.952892 | 0.906265 |
| 175 | 0.829487 | 0.867731 | 0.949548 | 0.906798 |
| 233 | 0.841323 | 0.922228 | 0.905573 | 0.913825 |
| 245 | 0.868004 | 0.919554 | 0.939334 | 0.929338 |
| 246 | 0.876301 | 0.978411 | 0.893579 | 0.934073 |
| Averge | 0.827353 | 0.8914132 | 0.921336 | 0.9051822 |

**Supplementary Table 11 Model-fitting results of PlantTPA**

| **NO.** | **Model** | **Model solution** | **Average coefficient** | **Result of**  **forecast model evaluation** | | |
| --- | --- | --- | --- | --- | --- | --- |
|  |  |  |  | **R^2^** | **MAPE** | **SD_APE_** |
| 1 | Linear model | TPA = at + b | a = 3.304E+03 | 0.733 | 0.921 | 2.223 |
|  |  |  | b =-7.181E+04 |  |  |  |
| 2 | Power model | TPA = at^b^ | a = 2.359E+03 | 0.726 | 1.377 | 3.629 |
|  |  |  | b = 1.086E+00 |  |  |  |
| 3 | Exponential model | TPA = ae^bt^ | a = 9.033E+04 | 0.615 | 1.876 | 5.157 |
|  |  |  | b = 1.015E-02 |  |  |  |
| 4 | Logarithm model | TPA = aln(bt) | a = 3.173E+05 | 0.829 | 0.504 | 1.021 |
|  |  |  | b = 2.731E-02 |  |  |  |
| 5 | Quadratic model | TPA = at^2^ + bt +c | a = -5.320E+01 | 0.944 | 0.642 | 2.032 |
|  |  |  | b = 1.403E+04 |  |  |  |
|  |  |  | c = -5.393E+05 |  |  |  |
| 6 | Gussian model | $TPA=ae^{{-(t-b)}^{2}/2c^{2}}$ | a = 4.151E+05 | 0.804 | 0.771 | 1.926 |
|  |  |  | b = 1.255E+02 |  |  |  |
|  |  |  | c = 4.357E+01 |  |  |  |

**Supplementary Table 12 Model-fitting results of PlantYpar**

| **NO.** | **Model** | **Model solution** | **coefficient** | **Result of**  **forecast model evaluation** | | |
| --- | --- | --- | --- | --- | --- | --- |
|  |  |  |  | **R^2^** | **MAPE** | **SD_APE_** |
| 1 | Linear model | ypar = at + b | a = 2.471E-03 | 0.511 | 69.9% | 0.638 |
|  |  |  | b = -7.844E-02 |  |  |  |
| 2 | Power model | ypar = at^b^ | a = -7.354E-01 | 0.559 | 100.7% | 0.986 |
|  |  |  | b = -1.070E+01 |  |  |  |
| 3 | Exponential model | ypar = ae^bt^ | a = 2.473E-02 | 0.722 | 60.6% | 0.551 |
|  |  |  | b = 2.029E-02 |  |  |  |
| 4 | Logarithm model | ypar = aln(bt) | a = 1.880E-01 | 0.389 | 79.5% | 0.739 |
|  |  |  | b = 8.077E-02 |  |  |  |
| 5 | Quadratic model | ypar = at^2^ + bt +c | a = 6.828E-05 | 0.916 | 23.4% | 0.213 |
|  |  |  | b = -1.090E-02 |  |  |  |
|  |  |  | c = 4.865E-01 |  |  |  |
| 6 | Gussian model | $ypar=ae^{{-(t-b)}^{2}/2c^{2}}$ | a = 3.067E-01 | 0.553 | 60.9% | 0.444 |
|  |  |  | b = 1.559E+02 |  |  |  |
|  |  |  | c = 4.409E+01 |  |  |  |

**Supplementary Table 13 Model-fitting results of PanicleTPA**

| **NO.** | **Model** | **Model solution** | **coefficient** | **Result of**  **forecast model evaluation** | | |
| --- | --- | --- | --- | --- | --- | --- |
|  |  |  |  | **R^2^** | **MAPE** | **SD_APE_** |
| 1 | Linear model | TPA = at + b | a = 6.222E+02 | 0.588 | 45.4% | 0.978 |
|  |  |  | b = -3.406E+04 |  |  |  |
| 2 | Power model | TPA = at^b^ | a = 2.592E+15 | 0.563 | 54.4% | 1.239 |
|  |  |  | b = 1.477E+00 |  |  |  |
| 3 | Exponential model | TPA = ae^bt^ | a = 1.840E+05 | 0.526 | 58.5% | 1.353 |
|  |  |  | b = 1.079E+05 |  |  |  |
| 4 | Logarithm model | TPA = aln(bt) | a = 8.268E+04 | 0.625 | 40.2% | 0.842 |
|  |  |  | b = 1.685E+00 |  |  |  |
| 5 | Quadratic model | TPA = at^2^ + bt +c | a = -2.367E+01 | 0.846 | 16.3% | 0.280 |
|  |  |  | b = 6.853E+03 |  |  |  |
|  |  |  | c = -4.445E+05 |  |  |  |
| 6 | Gussian model | $TPA=ae^{{-(t-b)}^{2}/2c^{2}}$ | a = 6.237E+04 | 0.704 | 43.9% | 0.969 |
|  |  |  | b = 1.444E+02 |  |  |  |
|  |  |  | c = 3.133E+01 |  |  |  |

**Supplementary Table 14 Model-fitting results of PanicleYpar**

| **NO.** | **Model** | **Model solution** | **coefficient** | **Result of**  **forecast model evaluation** | | |
| --- | --- | --- | --- | --- | --- | --- |
|  |  |  |  | **R^2^** | **MAPE** | **SD_APE_** |
| 1 | Linear model | ypar = at + b | a = 1.159E-02 | 0.875 | 0.269 | 0.337 |
|  |  |  | b = -1.032E+00 |  |  |  |
| 2 | Power model | ypar = at^b^ | a =7.300E+22 | 0.894 | 0.243 | 0.336 |
|  |  |  | b = 4.213E+00 |  |  |  |
| 3 | Exponential model | ypar = ae^bt^ | a = -8.768E-02 | 0.882 | 0.257 | 0.356 |
|  |  |  | b = -6.673E-02 |  |  |  |
| 4 | Logarithm model | ypar = aln(bt) | a = 1.432E+00 | 0.870 | 0.282 | 0.351 |
|  |  |  | b = 1.201E-02 |  |  |  |
| 5 | Quadratic model | ypar = at^2^ + bt +c | a = 6.000E-05 | 0.950 | 0.149 | 0.190 |
|  |  |  | b = -7.147E-03 |  |  |  |
|  |  |  | c = 3.734E-01 |  |  |  |
| 6 | Gussian model | $ypar=ae^{{-(t-b)}^{2}/2c^{2}}$ | a = 7.121E-01 | 0.904 | 0.214 | 0.223 |
|  |  |  | b = 1.555E+02 |  |  |  |
|  |  |  | c = 2.606E+01 |  |  |  |

**Supplementary Table 15 Statistical details of the coefficients of the selected model with plant-related traits for estimating yield**

| **Variable** | **Unstandardized coefficients** | | **Standardized coefficients** | **t** | **Sig.** |
| --- | --- | --- | --- | --- | --- |
|  | **Bata** | **Std.Error** | **Beta** |  |  |
| (Constant) | -18.744777 | 10.042 |  | -1.866647 | 0.065207 |
| PlantYPA | 0.000077 | 0.000077 | 0.478694 | 4.584 | 0.000015 |
| PlantH | 0.021101 | 0.007581 | 0.290663 | 2.783406 | 0.006555 |

**Supplementary Table 16 Statistical details of the coefficients of the selected model with panicle-related traits for estimating yield**

| **Variable** | **Unstandardized coefficients** | | **Standardized coefficients** | **t** | **Sig.** |
| --- | --- | --- | --- | --- | --- |
|  | **Bata** | **Std.Error** | **Beta** |  |  |
| (Constant) | 0.4098143 | 2.2202173 |  | 0.184583 | 0.8539669 |
| PanicleTPA | 0.0004091 | 0.0000318 | 0.8036524 | 12.8824892 | 3.17E-22 |

**Supplementary Table 17 Statistical details of the coefficients of the selected model with culm-related traits for estimating yield**

| **Variable** | **Unstandardized coefficients** | | **Standardized coefficients** | **t** | **Sig.** |
| --- | --- | --- | --- | --- | --- |
|  | **Bata** | **Std.Error** | **Beta** |  |  |
| (Constant) | 25.861 | 10.58 |  | 2.444 | 0.016 |
| culmH | 0.044 | 0.008 | 0.648 | 5.757 | 1.17E-07 |
| culmPHr | -59.993 | 18.008 | -0.375 | -3.331 | 0.001255 |

**Supplementary Table 18 Statistical details of the coefficients of the selected model with panicle growth-related traits for estimating yield**

| **Variable** | **Unstandardized coefficients** | | **Standardized coefficients** | **t** | **Sig.** |
| --- | --- | --- | --- | --- | --- |
|  | **Bata** | **Std.Error** | **Beta** |  |  |
| (Constant) | 11.91 | 1.654 |  | 7.2 | 1.84E-10 |
| PanicleYPAG | 0.006 | 0.001 | 0.451 | 6.399 | 7.11E-09 |
| PanicleYpar_a | 185027.257 | 41150.878 | 2.634 | 4.496 | 0.000021 |
| PanicleYpar_b | 578.966 | 157.831 | 2.147 | 3.668 | 0.000415 |

**Supplementary Table 19 Statistical details of the coefficients of the selected model with plant growth-related traits for estimating yield**

| **Variable** | **Unstandardized coefficients** | | **Standardized coefficients** | **t** | **Sig.** |
| --- | --- | --- | --- | --- | --- |
|  | **Bata** | **Std.Error** | **Beta** |  |  |
| (Constant) | -8.292288 | 5.151061 |  | -1.609821 | 0.111018 |
| PlantYPAG | 0.021111 | 0.003482 | 0.815295 | 6.062845 | 3.26E-08 |
| PlantHG | 1.400689 | 0.371702 | 0.285159 | 3.768311 | 0.000297 |
| PlantYparG | -5584.477209 | 1491.983313 | -0.423884 | -3.742989 | 0.000324 |
| PlantYpar_b | -879.927723 | 328.243751 | -0.317506 | -2.680714 | 0.00877 |

**Supplementary Table 20 Statistical details of the coefficients of the selected model with all i-traits for estimating yield**

| **Variable** | **Unstandardized coefficients** | | **Standardized coefficients** | **t** | **Sig.** |
| --- | --- | --- | --- | --- | --- |
|  | **Bata** | **Std.Error** | **Beta** |  |  |
| (Constant) | -6.948 | 5.84 |  | -1.19 | 0.238 |
| PanicleTPA | 0.000295 | 0.000037 | 0.579 | 7.99 | 6.35E-12 |
| PanicleYpar_a | -229830.634 | 89954.609 | -3.272 | -2.555 | 0.012 |
| PanicleYpar_b | -2137.907 | 608.723 | -7.93 | -3.512 | 0.001 |
| PanicleYpar | 42.107 | 6.561 | 0.684 | 6.418 | 7.80E-09 |
| PlantYparG | -5200.122 | 1007.693 | -0.395 | -5.16 | 0.000052 |
| PanicleYpar_c | -18.529 | 4.341 | -4.315 | -4.268 | 0.000052 |
| culmH | 0.013 | 0.004 | 0.198 | 3.223 | 0.002 |
| PlantHD | -0.082 | 0.041 | -0.15 | -2.004 | 0.048 |

**Supplementary Table 21 Trait groups of factors in the Mantel test analysis undertaken on the grain-related traits**

| **Variation** | **Traits group** | **Mantel statistic r** | **Significance** |
| --- | --- | --- | --- |
| grain-related traits | Plant-related traits | 0.5306 | 0.0001 |
|  | Panicle-related traits | 0.5011 | 0.0001 |
|  | Culm-related traits | 0.3528 | 0.0001 |
|  | Phenological traits | 0.2252 | 0.0001 |
|  | Panicle-growth-related traits | 0.4404 | 0.0001 |
|  | Plant-growth-related traits | 0.5239 | 0.0001 |

**Supplementary Table 22 QTLs detected by GWAS**

| **Chromosome** | **Start** | **End** | **Length** | **Trait** | **Significant SNPs in QTL** | **Lead SNP** | **minus log10 Pvalue** | **Note** |
| --- | --- | --- | --- | --- | --- | --- | --- | --- |
| 1 | 1389706 | 1618105 | 228399 | GP | 8 | 1518105 | 6.651072719 |  |
| 1 | 1389706 | 1618105 | 228399 | PlantYPAD | 6 | 1518105 | 6.36954506 |  |
| 1 | 1390351 | 1618105 | 227754 | PlantYparD | 6 | 1515276 | 6.07170199 |  |
| 1 | 1415276 | 1618105 | 202829 | PlantHD | 3 | 1515276 | 6.179280907 |  |
| 1 | 1418104 | 1618104 | 200000 | HS | 1 | 1518104 | 5.137062426 |  |
| 1 | 1418104 | 1618105 | 200001 | PlantSD | 2 | 1518105 | 6.103793138 |  |
| 1 | 2221731 | 2511112 | 289381 | PlantGPA | 72 | 2387065 | 6.606108973 |  |
| 1 | 2221731 | 2511112 | 289381 | PlantTPAG | 66 | 2387065 | 5.515743198 |  |
| 1 | 2221731 | 2511112 | 289381 | PlantTPA_c | 67 | 2387065 | 6.487570556 |  |
| 1 | 2280135 | 2506233 | 226098 | culmTPA | 47 | 2385897 | 5.37184287 |  |
| 1 | 2280135 | 2511112 | 230977 | PlantTPA | 49 | 2387065 | 5.888407614 |  |
| 1 | 2287065 | 2487085 | 200020 | PlantTPA_b | 3 | 2387065 | 5.633793019 |  |
| 1 | 5187949 | 5486114 | 298165 | PanicleYPAG | 25 | 5328176 | 5.344270142 |  |
| 1 | 6505084 | 6710613 | 205529 | PlantGPA | 2 | 6605084 | 5.797438155 |  |
| 1 | 6505084 | 6710613 | 205529 | PlantTPA | 2 | 6610613 | 4.993357437 |  |
| 1 | 6505084 | 6870885 | 365801 | PlantTPAG | 24 | 6605084 | 6.536508815 |  |
| 1 | 6505084 | 6870885 | 365801 | PlantTPA_c | 4 | 6605084 | 6.044208775 |  |
| 1 | 6505084 | 6710613 | 205529 | Time_PC1 | 2 | 6610613 | 6.388986661 |  |
| 1 | 6670885 | 6870885 | 200000 | culmH | 1 | 6770885 | 5.535214522 |  |
| 1 | 7247359 | 7635719 | 388360 | culmTPA | 12 | 7347359 | 5.226748445 |  |
| 1 | 7455477 | 7655477 | 200000 | Time_PC1 | 1 | 7555477 | 5.692429117 |  |
| 1 | 8217691 | 8417691 | 200000 | culmH | 1 | 8317691 | 5.480374896 |  |
| 1 | 9648105 | 9848105 | 200000 | PlantGPA | 1 | 9748105 | 5.319149878 |  |
| **Chromosome** | **Start** | **End** | **Length** | **Trait** | **Significant SNPs in QTL** | **Lead SNP** | **minus log10 Pvalue** | **Note** |
| 1 | 9648105 | 9848105 | 200000 | PlantTPA | 1 | 9748105 | 4.971149595 |  |
| 1 | 10473833 | 10673833 | 200000 | PlantTPAG | 1 | 10573833 | 4.815011039 |  |
| 1 | 13865481 | 14658672 | 793191 | PlantHG | 69 | 14146610 | 5.561145094 |  |
| 1 | 22251537 | 22552102 | 300565 | PlantHWr | 31 | 22375277 | 6.085151217 |  |
| 1 | 22252584 | 22459292 | 206708 | PlantHG | 2 | 22359292 | 5.359002013 |  |
| 1 | 23727869 | 24083881 | 356012 | PlantGPAD | 11 | 23974957 | 5.717228523 |  |
| 1 | 23728477 | 24001908 | 273431 | PlantHG | 4 | 23901818 | 5.548208469 |  |
| 1 | 24137869 | 24466725 | 328856 | PlantHD | 3 | 24278600 | 6.018145182 |  |
| 1 | 24162791 | 24367819 | 205028 | PlantSD | 2 | 24262791 | 5.759164214 |  |
| 1 | 24791946 | 25058106 | 266160 | PanicleYparG | 17 | 24935640 | 5.186028638 |  |
| 1 | 24791946 | 25048167 | 256221 | PlantSD | 10 | 24935640 | 6.04762933 |  |
| 1 | 24835640 | 25048167 | 212527 | PlantHD | 8 | 24935973 | 5.350809711 |  |
| 1 | 24835640 | 25048167 | 212527 | Time_PC2 | 8 | 24935640 | 5.68771798 |  |
| 1 | 25278966 | 25478966 | 200000 | PanicleYPAG | 1 | 25378966 | 5.61684618 |  |
| 1 | 27161090 | 27374523 | 213433 | PanicleTpar | 2 | 27261090 | 5.07023354 |  |
| 1 | 27161090 | 27374523 | 213433 | PlantGPAD | 2 | 27274523 | 5.642754105 |  |
| 1 | 40064106 | 40535577 | 471471 | PanicleYPAG | 18 | 40427304 | 5.964538226 |  |
| 2 | 0 | 136401 | 136401 | PanicleTpar | 1 | 36401 | 5.595485239 |  |
| 2 | 0 | 268036 | 268036 | PlantSD | 3 | 36401 | 5.88452944 |  |
| 2 | 52735 | 268036 | 215301 | HS | 2 | 152735 | 5.03219257 |  |
| 2 | 52735 | 268036 | 215301 | PlantGPA | 2 | 152735 | 5.162683211 |  |
| 2 | 52735 | 268036 | 215301 | PlantTPA | 2 | 152735 | 4.915547199 |  |
| 2 | 7955883 | 8214649 | 258766 | PlantYpar | 9 | 8071761 | 5.571286194 |  |
| 3 | 2588646 | 2807604 | 218958 | PlantYparD | 2 | 2688646 | 5.243433171 |  |
| **Chromosome** | **Start** | **End** | **Length** | **Trait** | **Significant SNPs in QTL** | **Lead SNP** | **minus log10 Pvalue** | **Note** |
| 3 | 2909920 | 3142028 | 232108 | PlantYparD | 2 | 3009920 | 5.326677586 |  |
| 3 | 13835932 | 14049671 | 213739 | AveLW | 7 | 13936021 | 6.971561846 |  |
| 3 | 16563167 | 17003634 | 440467 | AveLW | 46 | 16668465 | 6.275061332 | *GS3* |
| 3 | 16568465 | 16806516 | 238051 | AveGL | 3 | 16668465 | 7.193218301 | *GS3* |
| 3 | 16830967 | 17030967 | 200000 | AveGL | 1 | 16930967 | 6.900712904 |  |
| 3 | 22246687 | 22572216 | 325529 | PlantSD | 16 | 22459878 | 7.68825616 |  |
| 3 | 22325530 | 22572216 | 246686 | culmTPA | 8 | 22468399 | 5.901890137 |  |
| 3 | 22325530 | 22572216 | 246686 | PlantGPA | 8 | 22468399 | 6.195204118 |  |
| 3 | 22325530 | 22572216 | 246686 | PlantTPA | 8 | 22468399 | 5.728178646 |  |
| 3 | 22368399 | 22568399 | 200000 | culmH | 1 | 22468399 | 5.453553483 |  |
| 3 | 23296237 | 23555313 | 259076 | PlantTPAG | 2 | 23396237 | 5.457195945 |  |
| 3 | 23296237 | 23555313 | 259076 | PlantYparD | 2 | 23396237 | 4.974185109 |  |
| 3 | 23589071 | 23859784 | 270713 | PlantTPAG | 2 | 23759784 | 6.243800408 |  |
| 3 | 23659784 | 23859784 | 200000 | culmTPA | 1 | 23759784 | 5.789914453 |  |
| 3 | 23659784 | 23859784 | 200000 | GP | 1 | 23759784 | 6.462566289 |  |
| 3 | 23659784 | 23859784 | 200000 | HS | 1 | 23759784 | 5.612940004 |  |
| 3 | 23659784 | 23859784 | 200000 | PlantGPA | 1 | 23759784 | 6.23177553 |  |
| 3 | 23659784 | 23859784 | 200000 | PlantSD | 1 | 23759784 | 5.200122618 |  |
| 3 | 23659784 | 23859784 | 200000 | PlantTPA | 1 | 23759784 | 6.373279226 |  |
| 3 | 23659784 | 23859784 | 200000 | PlantTPAHr | 1 | 23759784 | 6.584968442 |  |
| 3 | 23659784 | 23859784 | 200000 | PlantYPAD | 1 | 23759784 | 7.315276194 |  |
| 3 | 23659784 | 23859784 | 200000 | PlantYparD | 1 | 23759784 | 7.014768486 |  |
| 3 | 23659784 | 23859784 | 200000 | PlantTPA_c | 1 | 23759784 | 5.971170781 |  |
| 3 | 23659784 | 23859784 | 200000 | Time_PC1 | 1 | 23759784 | 7.527231952 |  |
| **Chromosome** | **Start** | **End** | **Length** | **Trait** | **Significant SNPs in QTL** | **Lead SNP** | **minus log10 Pvalue** | **Note** |
| 3 | 23902993 | 24362598 | 459605 | culmTPA | 5 | 24262598 | 6.536161858 |  |
| 3 | 23902993 | 24362598 | 459605 | GP | 5 | 24068100 | 7.368874412 |  |
| 3 | 23902993 | 24362598 | 459605 | HS | 5 | 24262598 | 5.905370866 |  |
| 3 | 23902993 | 24362598 | 459605 | PlantGPA | 5 | 24262598 | 6.697967737 |  |
| 3 | 23902993 | 24362598 | 459605 | PlantSD | 5 | 24262598 | 5.571597082 |  |
| 3 | 23902993 | 24362598 | 459605 | PlantTPA | 6 | 24262598 | 6.943011578 |  |
| 3 | 23902993 | 24362598 | 459605 | PlantTPAG | 5 | 24262598 | 6.29421917 |  |
| 3 | 23902993 | 24362598 | 459605 | PlantTPAHr | 6 | 24262598 | 7.102105173 |  |
| 3 | 23902993 | 24362598 | 459605 | PlantYPAD | 5 | 24068100 | 8.237764565 |  |
| 3 | 23902993 | 24362598 | 459605 | PlantYparD | 5 | 24068100 | 7.646320965 |  |
| 3 | 23902993 | 24362598 | 459605 | PlantTPA_c | 5 | 24262598 | 6.331548649 |  |
| 3 | 23902993 | 24362598 | 459605 | Time_PC1 | 6 | 24262598 | 8.536857789 |  |
| 3 | 24162598 | 24362598 | 200000 | Organ_PC1 | 1 | 24262598 | 5.339376547 |  |
| 3 | 24162598 | 24362598 | 200000 | PlantTPA_b | 1 | 24262598 | 5.700975739 |  |
| 3 | 24768069 | 25016174 | 248105 | AveLW | 171 | 24868465 | 5.949054511 |  |
| 3 | 25565509 | 25774885 | 209376 | PlantGPA | 3 | 25665509 | 5.202233543 |  |
| 3 | 25565509 | 25774885 | 209376 | PlantTPA | 3 | 25665509 | 5.240144289 |  |
| 3 | 25565509 | 25776581 | 211072 | PlantTPA_c | 5 | 25674885 | 5.779680655 |  |
| 3 | 25574885 | 25781307 | 206422 | PlantTPAG | 5 | 25681293 | 5.152735597 |  |
| 3 | 25576497 | 25776581 | 200084 | PlantTPA_b | 2 | 25676581 | 5.537060088 |  |
| 3 | 30036923 | 30236923 | 200000 | PanicleYparG | 1 | 30136923 | 5.509736387 |  |
| 4 | 5117489 | 5317516 | 200027 | PanicleTpar | 3 | 5217489 | 5.239982833 |  |
| 4 | 7041977 | 7802145 | 760168 | GP | 34 | 7141977 | 6.241857044 |  |
| 4 | 7041977 | 8531281 | 1489304 | PanicleYparG | 46 | 8185796 | 6.096177808 |  |
| **Chromosome** | **Start** | **End** | **Length** | **Trait** | **Significant SNPs in QTL** | **Lead SNP** | **minus log10 Pvalue** | **Note** |
| 4 | 7041977 | 7802145 | 760168 | PlantYPAD | 34 | 7141977 | 6.384936075 |  |
| 4 | 7041977 | 8531281 | 1489304 | PlantYparD | 53 | 8185796 | 6.594037352 |  |
| 4 | 7041977 | 7250901 | 208924 | Time_PC2 | 2 | 7141977 | 6.206111115 |  |
| 4 | 7850904 | 8531281 | 680377 | GP | 13 | 8185796 | 6.13272633 |  |
| 4 | 7850904 | 8531281 | 680377 | PlantYPAD | 13 | 8185796 | 6.370741859 |  |
| 4 | 8085796 | 8285796 | 200000 | Time_PC2 | 1 | 8185796 | 6.180371013 |  |
| 4 | 8560735 | 9151840 | 591105 | GP | 8 | 8660735 | 6.473283599 |  |
| 4 | 8560735 | 9151840 | 591105 | PanicleYparG | 6 | 8660735 | 5.999559045 |  |
| 4 | 8560735 | 9151840 | 591105 | PlantYPAD | 7 | 8660735 | 6.732150689 |  |
| 4 | 8560735 | 9151840 | 591105 | PlantYparD | 8 | 8660735 | 6.831047331 |  |
| 4 | 8560735 | 8899842 | 339107 | Time_PC2 | 3 | 8660735 | 6.753684532 |  |
| 4 | 9309820 | 9511427 | 201607 | GP | 2 | 9411427 | 5.350639684 |  |
| 4 | 9309820 | 9770318 | 460498 | PanicleYparG | 4 | 9409820 | 5.357836438 |  |
| 4 | 9309820 | 9511427 | 201607 | PlantYPAD | 2 | 9411427 | 5.519915385 |  |
| 4 | 9309820 | 9770318 | 460498 | PlantYparD | 4 | 9411427 | 5.763371843 |  |
| 4 | 9570318 | 9770318 | 200000 | GP | 1 | 9670318 | 5.350639684 |  |
| 4 | 9570318 | 9770318 | 200000 | PlantYPAD | 1 | 9670318 | 5.519915385 |  |
| 4 | 9826810 | 10205016 | 378206 | GP | 4 | 10083758 | 7.765186445 |  |
| 4 | 9826810 | 10205016 | 378206 | PanicleYparG | 4 | 10083758 | 5.746437546 |  |
| 4 | 9826810 | 10205016 | 378206 | PlantYPAD | 4 | 10083758 | 8.189637575 |  |
| 4 | 9826810 | 10205016 | 378206 | PlantYparD | 4 | 10083758 | 8.165771202 |  |
| 4 | 9983758 | 10183758 | 200000 | Time_PC2 | 1 | 10083758 | 7.466275822 |  |
| 4 | 10382634 | 11052431 | 669797 | GP | 5 | 10952431 | 5.974112737 |  |
| 4 | 10382634 | 11052431 | 669797 | PlantYPAD | 5 | 10952431 | 6.17849032 |  |
| **Chromosome** | **Start** | **End** | **Length** | **Trait** | **Significant SNPs in QTL** | **Lead SNP** | **minus log10 Pvalue** | **Note** |
| 4 | 10382634 | 11056244 | 673610 | PlantYparD | 9 | 10952431 | 6.711062744 |  |
| 4 | 10532478 | 11056244 | 523766 | PanicleYparG | 5 | 10632478 | 5.404014315 |  |
| 4 | 10673672 | 10873672 | 200000 | PanicleYPAG | 1 | 10773672 | 5.024408449 |  |
| 4 | 10852431 | 11052431 | 200000 | Time_PC2 | 1 | 10952431 | 6.518896271 |  |
| 4 | 11117903 | 11317903 | 200000 | GP | 1 | 11217903 | 5.840939337 |  |
| 4 | 11117903 | 11317903 | 200000 | PlantYPAD | 1 | 11217903 | 6.323056845 |  |
| 4 | 11117903 | 11317903 | 200000 | PlantYparD | 1 | 11217903 | 6.473646781 |  |
| 4 | 11117903 | 11317903 | 200000 | Time_PC2 | 1 | 11217903 | 6.096787269 |  |
| 4 | 11456351 | 11698520 | 242169 | PanicleYparG | 2 | 11598520 | 5.809458575 |  |
| 4 | 11498520 | 11698520 | 200000 | GP | 1 | 11598520 | 5.142712124 |  |
| 4 | 11498520 | 11698520 | 200000 | PlantYparD | 1 | 11598520 | 5.281392024 |  |
| 4 | 11498520 | 11698520 | 200000 | Time_PC2 | 1 | 11598520 | 6.841267545 |  |
| 4 | 12434847 | 12634847 | 200000 | GP | 1 | 12534847 | 5.683637046 |  |
| 4 | 12434847 | 12634847 | 200000 | PanicleYparG | 1 | 12534847 | 5.865011673 |  |
| 4 | 12434847 | 12634847 | 200000 | PlantYPAD | 1 | 12534847 | 6.023556987 |  |
| 4 | 12434847 | 12634847 | 200000 | PlantYparD | 1 | 12534847 | 6.460949073 |  |
| 4 | 12434847 | 12634847 | 200000 | Time_PC2 | 1 | 12534847 | 6.868420583 |  |
| 4 | 16528770 | 16765718 | 236948 | culmGpar | 2 | 16628770 | 5.986763352 |  |
| 4 | 16528770 | 16784918 | 256148 | culmYpar | 8 | 16628770 | 5.98676729 |  |
| 4 | 28722991 | 29278559 | 555568 | PlantYparD | 17 | 28822991 | 5.389135942 |  |
| 4 | 29613192 | 29836892 | 223700 | PlantGPAD | 2 | 29713192 | 6.557802051 |  |
| 4 | 31864331 | 32064331 | 200000 | PlantHD | 1 | 31964331 | 5.549170971 |  |
| 5 | 1217428 | 1417428 | 200000 | PlantH | 1 | 1317428 | 5.702758863 |  |
| 5 | 5271529 | 5478981 | 207452 | AveLW | 28 | 5373841 | 5.795171913 | *GSE5* |
| **Chromosome** | **Start** | **End** | **Length** | **Trait** | **Significant SNPs in QTL** | **Lead SNP** | **minus log10 Pvalue** | **Note** |
| 6 | 19702 | 219702 | 200000 | PlantTPAG | 1 | 119702 | 5.675881415 |  |
| 6 | 19702 | 219702 | 200000 | PlantTPA_b | 1 | 119702 | 5.496998011 |  |
| 6 | 19702 | 219702 | 200000 | PlantTPA_c | 1 | 119702 | 5.430016586 |  |
| 6 | 19702 | 219702 | 200000 | Time_PC1 | 1 | 119702 | 5.496245193 |  |
| 6 | 416127 | 720214 | 304087 | HS | 4 | 516127 | 5.186789568 |  |
| 6 | 416127 | 772041 | 355914 | PlantGPA | 5 | 516127 | 6.380602684 |  |
| 6 | 416127 | 720214 | 304087 | PlantGPAD | 4 | 516127 | 7.565028448 |  |
| 6 | 416127 | 720214 | 304087 | PlantSD | 4 | 516127 | 7.443728903 |  |
| 6 | 416127 | 772041 | 355914 | PlantTPA | 5 | 516127 | 6.089063306 |  |
| 6 | 416127 | 772041 | 355914 | PlantTPAG | 5 | 516127 | 6.028998467 |  |
| 6 | 416127 | 720214 | 304087 | PlantTPAHr | 4 | 516127 | 5.278210964 |  |
| 6 | 416127 | 772041 | 355914 | PlantTPA_b | 5 | 516127 | 6.309125189 |  |
| 6 | 416127 | 772041 | 355914 | PlantTPA_c | 5 | 516127 | 6.822427762 |  |
| 6 | 416127 | 772041 | 355914 | Time_PC1 | 5 | 516127 | 8.599347763 |  |
| 6 | 838264 | 1069936 | 231672 | PlantTPAG | 2 | 938264 | 4.852847376 |  |
| 6 | 838264 | 1069936 | 231672 | PlantTPA_c | 2 | 938264 | 5.748903813 |  |
| 6 | 838264 | 1069936 | 231672 | Time_PC1 | 2 | 938264 | 6.841784402 |  |
| 6 | 1333921 | 1533921 | 200000 | PlantGPAD | 1 | 1433921 | 5.269634244 |  |
| 6 | 1546592 | 1793474 | 246882 | PlantGPAD | 2 | 1646592 | 7.066231952 |  |
| 6 | 3022712 | 3222712 | 200000 | PlantYparD | 1 | 3122712 | 5.248495264 |  |
| 6 | 5776838 | 5976838 | 200000 | PlantGPAD | 1 | 5876838 | 5.348077241 |  |
| 6 | 10734425 | 10986027 | 251602 | PlantGPA | 7 | 10886027 | 6.569890414 |  |
| 6 | 10734425 | 10986027 | 251602 | PlantTPA | 6 | 10886027 | 6.38539241 |  |
| 6 | 10734425 | 10986027 | 251602 | PlantTPAG | 2 | 10886027 | 6.361884906 |  |
| **Chromosome** | **Start** | **End** | **Length** | **Trait** | **Significant SNPs in QTL** | **Lead SNP** | **minus log10 Pvalue** | **Note** |
| 6 | 10786027 | 10986027 | 200000 | Organ_PC1 | 1 | 10886027 | 5.166012905 |  |
| 6 | 10786027 | 10986027 | 200000 | PlantTPA_c | 1 | 10886027 | 5.718726379 |  |
| 6 | 10786027 | 10986027 | 200000 | Time_PC1 | 1 | 10886027 | 5.541795335 |  |
| 6 | 11491821 | 11691821 | 200000 | PlantGPAD | 1 | 11591821 | 5.336126336 |  |
| 6 | 13647269 | 13849223 | 201954 | PanicleYPAG | 2 | 13749223 | 5.33991543 |  |
| 6 | 13684330 | 13902546 | 218216 | Time_PC1 | 3 | 13784330 | 5.447599291 |  |
| 6 | 13976684 | 14392421 | 415737 | PanicleYPAG | 3 | 14208906 | 5.660962032 |  |
| 6 | 14436835 | 14636835 | 200000 | PanicleYPAG | 1 | 14536835 | 5.26829791 |  |
| 6 | 14685459 | 14977364 | 291905 | PanicleYPAG | 2 | 14785459 | 5.412498748 |  |
| 6 | 15009915 | 15209915 | 200000 | PanicleYPAG | 1 | 15109915 | 5.26829791 |  |
| 6 | 15213627 | 15413627 | 200000 | PanicleYPAG | 1 | 15313627 | 5.26829791 |  |
| 6 | 21264321 | 21734586 | 470265 | PlantGPAD | 751 | 21528829 | 8.695744527 |  |
| 6 | 25899380 | 26136848 | 237468 | PlantSD | 3 | 25999380 | 5.097733121 |  |
| 7 | 1673393 | 1873393 | 200000 | PlantH | 1 | 1773393 | 5.213313031 |  |
| 7 | 2857519 | 3057519 | 200000 | GP | 1 | 2957519 | 5.556357662 |  |
| 7 | 2857519 | 3057519 | 200000 | HS | 1 | 2957519 | 5.490876319 |  |
| 7 | 2857519 | 3057519 | 200000 | PanicleTpar | 1 | 2957519 | 5.015319056 |  |
| 7 | 2857519 | 3057519 | 200000 | PlantYPAD | 1 | 2957519 | 5.416100549 |  |
| 7 | 2857519 | 3057519 | 200000 | PlantYparD | 1 | 2957519 | 5.144217689 |  |
| 7 | 3067785 | 3400456 | 332671 | PlantTPAG | 11 | 3172922 | 5.534358049 |  |
| 7 | 3109243 | 3357943 | 248700 | GP | 6 | 3209243 | 7.079444315 |  |
| 7 | 3109243 | 3357943 | 248700 | PlantYPAD | 6 | 3209243 | 7.024850416 |  |
| 7 | 3109243 | 3357943 | 248700 | PlantYparD | 6 | 3209243 | 7.438564537 |  |
| 7 | 4511059 | 4731744 | 220685 | GP | 2 | 4611059 | 6.530075587 |  |
| **Chromosome** | **Start** | **End** | **Length** | **Trait** | **Significant SNPs in QTL** | **Lead SNP** | **minus log10 Pvalue** | **Note** |
| 7 | 4511059 | 4731744 | 220685 | PlantYPAD | 2 | 4611059 | 6.407468327 |  |
| 7 | 4511059 | 4711059 | 200000 | PlantYparD | 1 | 4611059 | 6.08642134 |  |
| 7 | 4609186 | 4809186 | 200000 | PanicleTPAD | 1 | 4709186 | 5.670851346 |  |
| 7 | 4801042 | 5001042 | 200000 | PlantH | 1 | 4901042 | 5.18089687 |  |
| 7 | 5033458 | 5273610 | 240152 | PlantH | 2 | 5173610 | 5.204530317 |  |
| 7 | 8640726 | 8875268 | 234542 | culmTPA | 4 | 8740726 | 5.044903887 |  |
| 7 | 8662552 | 8862552 | 200000 | culmH | 1 | 8762552 | 5.595330695 |  |
| 7 | 8662552 | 8894750 | 232198 | PlantHD | 2 | 8762552 | 5.71999055 |  |
| 7 | 9176029 | 10107598 | 931569 | PlantHD | 14 | 9654913 | 6.119026961 |  |
| 7 | 9176029 | 10107598 | 931569 | PlantTPAG | 13 | 9360714 | 5.248283398 |  |
| 7 | 9176029 | 10107598 | 931569 | Time_PC1 | 18 | 9654913 | 7.645745348 |  |
| 7 | 9260714 | 9460714 | 200000 | PlantGPA | 1 | 9360714 | 5.20058902 |  |
| 7 | 9260714 | 9460714 | 200000 | PlantTPA | 1 | 9360714 | 5.317622893 |  |
| 7 | 9260714 | 9460714 | 200000 | PlantYparD | 1 | 9360714 | 5.590531217 |  |
| 7 | 9260714 | 9460714 | 200000 | Organ_PC1 | 1 | 9360714 | 5.560209018 |  |
| 7 | 9554913 | 9888346 | 333433 | PlantGPA | 2 | 9654913 | 5.33481713 |  |
| 7 | 9554913 | 9888346 | 333433 | PlantTPA | 2 | 9654913 | 5.174920278 |  |
| 7 | 9554913 | 9754913 | 200000 | PlantTPAHr | 1 | 9654913 | 5.308654941 |  |
| 7 | 10980193 | 11443349 | 463156 | PlantHD | 6 | 11080397 | 5.672949867 |  |
| 7 | 10980193 | 11180397 | 200204 | Time_PC1 | 2 | 11080397 | 5.85607114 |  |
| 7 | 10980397 | 11180397 | 200000 | PlantTPAG | 1 | 11080397 | 4.695387493 |  |
| 7 | 11556917 | 11779193 | 222276 | PlantHD | 5 | 11656917 | 6.358788638 |  |
| 7 | 11765100 | 12055332 | 290232 | Time_PC1 | 2 | 11865100 | 5.674357056 |  |
| 7 | 11827649 | 12027649 | 200000 | PlantHD | 1 | 11927649 | 5.402874313 |  |
| **Chromosome** | **Start** | **End** | **Length** | **Trait** | **Significant SNPs in QTL** | **Lead SNP** | **minus log10 Pvalue** | **Note** |
| 7 | 12016185 | 12216185 | 200000 | Organ_PC1 | 1 | 12116185 | 5.118905335 |  |
| 7 | 12228490 | 12428490 | 200000 | Time_PC1 | 1 | 12328490 | 5.6077442 |  |
| 7 | 12239271 | 12512091 | 272820 | PlantHD | 3 | 12412091 | 5.871469861 |  |
| 7 | 12768029 | 13060860 | 292831 | PlantHD | 5 | 12892777 | 6.228369897 |  |
| 7 | 15923071 | 16372385 | 449314 | PlantH | 12 | 16272385 | 6.531062739 |  |
| 7 | 16145580 | 16345580 | 200000 | PlantYparD | 1 | 16245580 | 5.584540483 |  |
| 7 | 16172385 | 16606086 | 433701 | culmTPA | 6 | 16448256 | 5.182863121 |  |
| 7 | 16777082 | 17164178 | 387096 | PlantH | 3 | 17025400 | 6.314027533 |  |
| 7 | 16777082 | 17164178 | 387096 | PlantTPAG | 3 | 16877082 | 5.659692702 |  |
| 7 | 20507571 | 20818968 | 311397 | GP | 14 | 20640793 | 5.698705378 |  |
| 7 | 20507571 | 20818968 | 311397 | PlantYparD | 16 | 20640793 | 6.435376687 |  |
| 7 | 21082706 | 21342452 | 259746 | GP | 91 | 21219853 | 6.281514022 |  |
| 7 | 21082706 | 21427914 | 345208 | PlantYPAD | 43 | 21238397 | 6.189834777 |  |
| 7 | 21082706 | 21342452 | 259746 | PlantYparD | 93 | 21209740 | 6.468551403 |  |
| 7 | 25459074 | 25659074 | 200000 | PlantYpar | 1 | 25559074 | 5.022170711 |  |
| 7 | 29119443 | 29319443 | 200000 | PlantHD | 1 | 29219443 | 5.20125651 |  |
| 8 | 9904553 | 10170104 | 265551 | PlantGPAD | 16 | 10035299 | 7.176201047 |  |
| 8 | 16682444 | 16882645 | 200201 | AveLW | 16 | 16782444 | 5.65607548 |  |
| 8 | 20344028 | 20544028 | 200000 | PlantYPAD | 1 | 20444028 | 5.299222797 |  |
| 8 | 25931621 | 26452710 | 521089 | PlantGPAD | 8 | 26172877 | 6.730721651 |  |
| 8 | 26644653 | 26855841 | 211188 | PlantGPAD | 2 | 26744653 | 5.650420613 |  |
| 8 | 27525722 | 27725722 | 200000 | PlantGPAD | 1 | 27625722 | 5.490740691 |  |
| 9 | 7812197 | 8012197 | 200000 | PanicleYpar_c | 1 | 7912197 | 6.089519509 |  |
| 9 | 12468549 | 12668549 | 200000 | GP | 1 | 12568549 | 5.307015109 |  |
| **Chromosome** | **Start** | **End** | **Length** | **Trait** | **Significant SNPs in QTL** | **Lead SNP** | **minus log10 Pvalue** | **Note** |
| 9 | 12468549 | 12668549 | 200000 | PlantYparD | 1 | 12568549 | 5.125078671 |  |
| 9 | 14418043 | 14679646 | 261603 | GP | 6 | 14579646 | 5.586546035 |  |
| 9 | 14475881 | 14679646 | 203765 | PlantYparD | 2 | 14579646 | 5.295652422 |  |
| 9 | 14785605 | 15038668 | 253063 | GP | 2 | 14938668 | 6.091247945 |  |
| 9 | 14785605 | 15038668 | 253063 | PlantYparD | 2 | 14938668 | 5.843627778 |  |
| 9 | 14838668 | 15038668 | 200000 | PlantYPAD | 1 | 14938668 | 5.512782052 |  |
| 9 | 16103126 | 16416727 | 313601 | GP | 3 | 16316727 | 5.26961345 |  |
| 9 | 16103126 | 16371846 | 268720 | PlantHD | 2 | 16203126 | 6.264755352 |  |
| 9 | 16103126 | 16371846 | 268720 | PlantHG | 2 | 16203126 | 5.331181563 |  |
| 9 | 16103126 | 16371846 | 268720 | PlantSD | 2 | 16203126 | 5.716271435 |  |
| 9 | 16103126 | 16416727 | 313601 | PlantYparD | 3 | 16316727 | 5.737435249 |  |
| 9 | 16103126 | 16371846 | 268720 | Time_PC2 | 2 | 16203126 | 5.734360038 |  |
| 9 | 16216727 | 16615203 | 398476 | PlantGPA | 4 | 16496805 | 5.524668951 |  |
| 9 | 16216727 | 16615203 | 398476 | PlantTPA | 4 | 16496805 | 5.36860931 |  |
| 9 | 16216727 | 16615203 | 398476 | Time_PC1 | 4 | 16498834 | 6.195723542 |  |
| 9 | 16396805 | 16615203 | 218398 | PlantTPAG | 2 | 16496805 | 4.703628623 |  |
| 9 | 16396805 | 16615203 | 218398 | PlantTPA_c | 2 | 16496805 | 5.565794834 |  |
| 9 | 17899987 | 18163139 | 263152 | PlantHD | 11 | 18063139 | 6.476463792 |  |
| 9 | 17899987 | 18163139 | 263152 | PlantHG | 11 | 18063139 | 6.108779896 |  |
| 9 | 17899987 | 18163139 | 263152 | PlantSD | 10 | 18030519 | 6.011862665 |  |
| 9 | 17899987 | 18161794 | 261807 | Time_PC2 | 8 | 17999987 | 5.473875295 |  |
| 9 | 18520146 | 18894595 | 374449 | PlantHG | 25 | 18744887 | 5.90156529 |  |
| 9 | 18644887 | 18894595 | 249708 | PlantHD | 2 | 18744887 | 5.805383389 |  |
| 9 | 19263551 | 19463551 | 200000 | PlantHG | 1 | 19363551 | 5.056269228 |  |
| **Chromosome** | **Start** | **End** | **Length** | **Trait** | **Significant SNPs in QTL** | **Lead SNP** | **minus log10 Pvalue** | **Note** |
| 9 | 20406875 | 20753687 | 346812 | PlantHG | 4 | 20506875 | 5.056269228 |  |
| 9 | 20780774 | 20992064 | 211290 | PlantHG | 2 | 20892064 | 5.220490883 |  |
| 9 | 21304799 | 21718116 | 413317 | PlantHG | 37 | 21567668 | 5.417615727 |  |
| 9 | 21330238 | 21530238 | 200000 | Time_PC2 | 1 | 21430238 | 5.409295359 |  |
| 10 | 5327511 | 5920687 | 593176 | PanicleYpar_c | 183 | 5427511 | 6.153408226 |  |
| 10 | 6351002 | 6551002 | 200000 | AveLW | 1 | 6451002 | 5.203170896 |  |
| 10 | 11168713 | 11368713 | 200000 | AveLW | 1 | 11268713 | 5.304582404 |  |
| 10 | 11741132 | 11945617 | 204485 | AveLW | 2 | 11841132 | 5.624434933 |  |
| 11 | 6218755 | 6418755 | 200000 | PlantH | 1 | 6318755 | 6.11288305 |  |
| 11 | 6218755 | 6418755 | 200000 | PlantTPAG | 1 | 6318755 | 5.547210911 |  |
| 11 | 7782195 | 7995647 | 213452 | PlantH | 8 | 7890678 | 6.11288305 |  |
| 11 | 7782195 | 7995647 | 213452 | PlantTPAG | 8 | 7890678 | 5.547210911 |  |
| 11 | 10333761 | 10758603 | 424842 | AveLW | 189 | 10565022 | 6.903407064 |  |
| 11 | 17844389 | 18110832 | 266443 | HS | 2 | 17944389 | 5.414615166 |  |
| 11 | 18073357 | 18535996 | 462639 | GP | 9 | 18399752 | 8.55945956 |  |
| 11 | 18183673 | 18535996 | 352323 | PlantYparD | 8 | 18399752 | 7.021800606 |  |
| 11 | 18299752 | 18535996 | 236244 | PlantYPAD | 2 | 18399752 | 7.454265024 |  |
| 12 | 1448582 | 1648582 | 200000 | W | 1 | 1548582 | 6.36996872 |  |
| 12 | 1623056 | 1823056 | 200000 | SF | 1 | 1723056 | 10.25291707 |  |
| 12 | 1901071 | 2101071 | 200000 | W | 1 | 2001071 | 7.573848257 |  |
| 12 | 2356980 | 2556980 | 200000 | SF | 1 | 2456980 | 11.06696058 |  |
| 12 | 22328329 | 22528329 | 200000 | PlantH | 1 | 22428329 | 5.478333886 |  |
